# Comprehensive management of gestational diabetes mellitus: practical efficacy of exercise therapy and sustained intervention strategies

**DOI:** 10.3389/fendo.2024.1347754

**Published:** 2024-10-03

**Authors:** Hao Xu, Renyi Liu

**Affiliations:** School of Physical Education, China University of Geosciences, Wuhan, China

**Keywords:** gestational diabetes mellitus, type 2 diabetes, exercise, lifestyle, meta-analysis

## Abstract

**Background:**

Gestational Diabetes Mellitus (GDM) affects 14.0% of pregnancies globally, with a 35% post-pregnancy relapse and a 60% risk of Type 2 Diabetes (T2D) within 5-10 years. Challenges in long-term management, especially postpartum, include adherence and follow-up difficulties.

**Methods:**

This study, based on a systematic review and meta-analysis, examined the practical effects of exercise therapy in the prevention, treatment, and prevention of progression from Gestational Diabetes Mellitus (GDM) to Type 2 Diabetes (T2D). Relevant research and clinical practices were retrieved from six major databases (PubMed, Scopus, Web of Science, Cochrane Library, MEDLINE, Science Direct). After analyzing the intervention effects of exercise therapy at different stages, factors favorably influencing the effectiveness of exercise intervention were identified during the more effective stages. Finally, a long-term and efficient exercise implementation plan for the comprehensive management of GDM was proposed.

**Results:**

In GDM prevention, exercise reduced the post-intervention risk by 37% compared to the control group (Relative Risk (RR)=0.63; 95% Confidence Interval (CI): 0.54 to 0.72; p=0.01). Studies on GDM treatment showed improved glucose control in the exercise group post-intervention (Mean Difference (MD)=-0.10; 95% CI: -0.16 to -0.04; p=0.04/MD=-0.27; 95% CI: -0.36 to -0.19; p<0.0001). However, exercise therapy didn’t significantly affect the incidence of T2D post-GDM (RR=0.88; 95% CI: 0.69 to 1.11; p=0.39) due to challenges in quantified exercise prescriptions and the complexity of postpartum programs.

**Conclusion:**

To enhance exercise therapy effectiveness in GDM management, the study recommends adopting an integrated model emphasizing personalized pregnancy plans, postpartum strategies, and long-term support. Leveraging frequent healthcare contact during pregnancy can establish and sustain exercise habits, fostering a lifelong pattern. While the study acknowledges limitations, this approach holds potential for improving glycemic metabolism and developing healthy exercise habits in subsequent generations. Future research should include longer follow-ups to validate the practical efficacy of this approach in preventing T2D after GDM.

**Systematic review registration:**

https://www.crd.york.ac.uk/prospero, identifier CRD42023463617.

## 1 Introduction

Gestational Diabetes Mellitus (GDM) is a metabolic disorder that commonly occurs during pregnancy and is typically screened between weeks 24~28 through a 75g Oral Glucose Tolerance Test (OGTT). Currently, the global incidence of GDM is approximately 14.0% (95% CI: 13.97-14.04%) (1).Without intervention, GDM patients have a recurrence rate of 35% in subsequent pregnancies (95% CI, 25.5-44.5%) (2), and 60% of GDM patients may develop Type 2 Diabetes (T2D) within 5-10 years postpartum (3). Women with a history of GDM have nearly a 10-fold increased risk of developing T2D compared to those with normal glucose pregnancies (4).

Diabetes prevention programs suggest that lifestyle interventions can reduce the risk of developing T2D by 34% within 10 years (5). Lifestyle interventions, as non-pharmacological approaches, typically include exercise and dietary interventions, with exercise intervention being the most challenging to maintain. In studies on exercise therapy for preventing GDM, pregnant women demonstrate high adherence, significantly reducing the incidence of GDM (6). However, in studies on exercise therapy for treating GDM, there is often a decrease in adherence among pregnant women. Specifically, in research on exercise therapy in the post-GDM prognosis stage, only one-third of individuals engage in physical activity (7). A systematic review suggests that while physical activity can reduce the risk of T2D, exercise intervention measures in the post-GDM stage largely fail to modify their physical activity behavior (8). This suggests that the practical effectiveness of exercise intervention in post-GDM management may be less than theoretical.

The aforementioned studies on exercise therapy management of GDM collectively contribute to the comprehensive management of GDM, encompassing prevention, treatment, patient prognosis, and long-term health. To enhance the intervention effectiveness of exercise therapy in the comprehensive management of GDM, it is essential to address the issue of inadequate adherence to exercise in the post-GDM prognosis stage.

Focusing on these issues, this study aims to explore the practical effects of exercise therapy in the prevention, treatment, and prevention of GDM progression to Type 2 Diabetes. We will integrate the latest research and clinical practices, conducting meta-analyses to understand the varying intervention effects of exercise therapy in different stages of GDM –prevention, treatment, and post-prognosis. Subsequently, starting from the stages where exercise intervention shows better results, we will analyze and summarize favorable factors influencing exercise intervention effectiveness. Finally, we will discuss how to leverage these factors to improve women’s adherence to exercise in the post-GDM prognosis stage, ensuring maximum health benefits. Our aim is to maximize the pivotal role of exercise therapy in managing GDM, enhancing women’s metabolic health, and mitigating the risk of future T2D and other chronic diseases. We envisage offering valuable insights for the fields of obstetrics and gynecology, as well as the healthcare system at large.

## 2 Method

Our study protocol was registered with the PROSPERO (Registration number: CRD42023463617) and was developed according to PRISMA and the Cochrane Collaboration Handbook (9, 10).

### 2.1 Search strategy

Search of the PubMed, Scopus, Web of Science, Cochrane Library, MEDLINE, Science Direct (Elsevier) from inception to November 2023 for randomized controlled trials (RCTs) aimed at assessing the effect of exercise interventions on GDM and Type 2 Diabetes After GDM. We conducted searches for all relevant subject terms and free-text keywords related to exercise, randomized controlled trials, GDM, and type 2 diabetes after GDM, and then formulated search strategies based on the retrieval requirements of different databases. See **Supplementary File 1** for specific search strategies.

### 2.2 Inclusion and exclusion criteria

#### 2.2.1 Inclusion criteria

Participants: Normal pregnant women (studies on GDM prevention), pregnant women with gestational diabetes mellitus (GDM) (studies on GDM treatment), or women with a history of GDM (studies on GDM prognosis). Intervention: Exercise intervention in the experimental group. Control: Peripartum women receiving routine care or other therapies not involving physical activity intervention served as the control group. Outcome Measures: The primary outcome measures include the incidence of GDM/T2D after GDM and the results of the 75-gram oral glucose tolerance test (75g-OGTT). Study Design: Randomized controlled trials (RCTs) as the analysis type in the literature.

#### 2.2.2 Exclusion criteria

Participants: Pregnant women with a history of diabetes or a family history of diabetes, and those with contraindications to exercise. Intervention: Studies where the intervention does not include exercise. Control: Studies lacking information on the control group. Data: Studies with incomplete data or studies from which valid data cannot be extracted. Study Types: Conference reports, protocols, case reports, reviews, and editorial materials.

### 2.3 Quality assessment and data extraction

**Figure 1:**
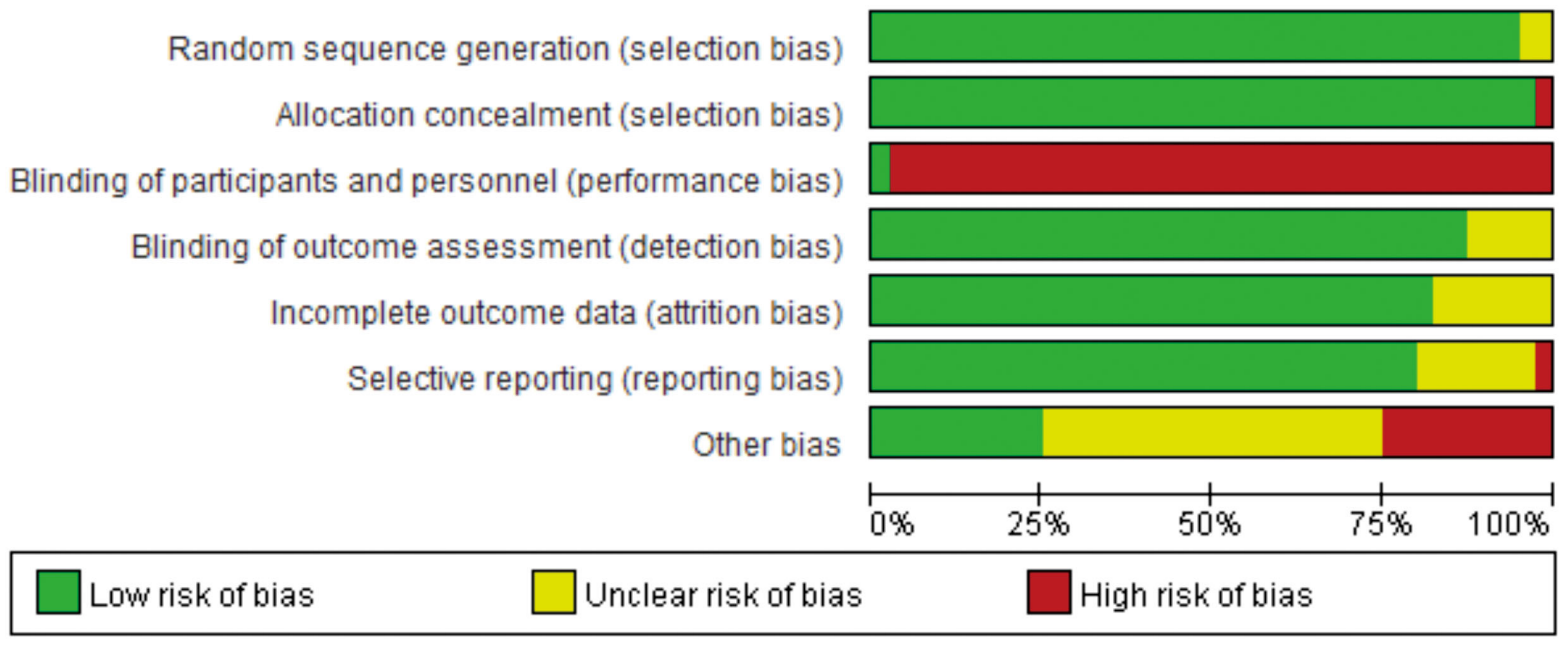
Bias risk assessment of included studies.

According to the preliminary risk assessment guidelines recommended by the Cochrane Collaboration, the following parameters were considered in the analysis: adequate random sequence generation, allocation concealment, blinding of participants and personnel, blinding of outcome assessment, incomplete outcome data, selective reporting and other bias (**Figure 1**). In the following steps, we integrated the relevant data from all the studies, mainly categorized into three groups, namely, outcome indicators of exercise therapy in the prevention, treatment, and post-prognosis stages of GDM. These indicators include GDM incidence, 75g-OGTT results, and Type 2 Diabetes (T2D) incidence. Subsequently, we conducted independent meta-analyses for these three categories of data to determine whether there are differences in the intervention effects of exercise management for GDM across these three stages.

### 2.4 Statistical analyses

Using RevMan 5.4 software, the mean and standard deviation of the 75g oral glucose tolerance test (75g-OGTT) in the experimental and control groups after exercise intervention, as well as the incidence data of gestational diabetes mellitus (GDM) and type 2 diabetes after GDM, were analyzed. The 75g-OGTT is a clinical laboratory examination used to assess an individual’s blood sugar metabolism. This test involves the oral consumption of a 75-gram glucose solution while the individual is in a fasting state. Subsequently, blood glucose levels are measured at different time intervals, typically including 0 hours (fasting), 1 hour, and 2 hours after glucose ingestion.

The above content falls into the category of continuous data, and we calculated the effect size (MD) and its 95% confidence interval for the experimental and control groups in each study. By summarizing the results from all randomized controlled trials (RCTs) included in the meta-analysis, we could determine whether there was an improvement in fasting blood glucose and blood glucose 2 hours after ingestion. This depended on whether there was a significant difference in the outcomes between the experimental and control groups. If MD was positive, it indicated that the mean of the experimental group was higher than that of the control group, and if MD was negative, it indicated that the control group’s mean was higher than that of the experimental group.

Regarding the incidence rates of GDM and type 2 diabetes after GDM, the data are dichotomous. We used Relative Risk (RR) to compare the differences in the incidence rates of gestational diabetes and type 2 diabetes after GDM between the exercise group and the control group. If RR equaled 1, then the incidence rates in both groups were equal; if RR was greater than 1, it indicated a higher incidence rate in the exercise group, and if RR was less than 1, it indicated a lower incidence rate in the exercise group. Whether it was continuous or dichotomous outcomes, if I^2^ was less than or equal to 50%, it indicated low heterogeneity. Conversely, if I^2^ was greater than 50%, it indicated high heterogeneity.

## 3 Results

### 3.1 Search results

**Figure 2:**
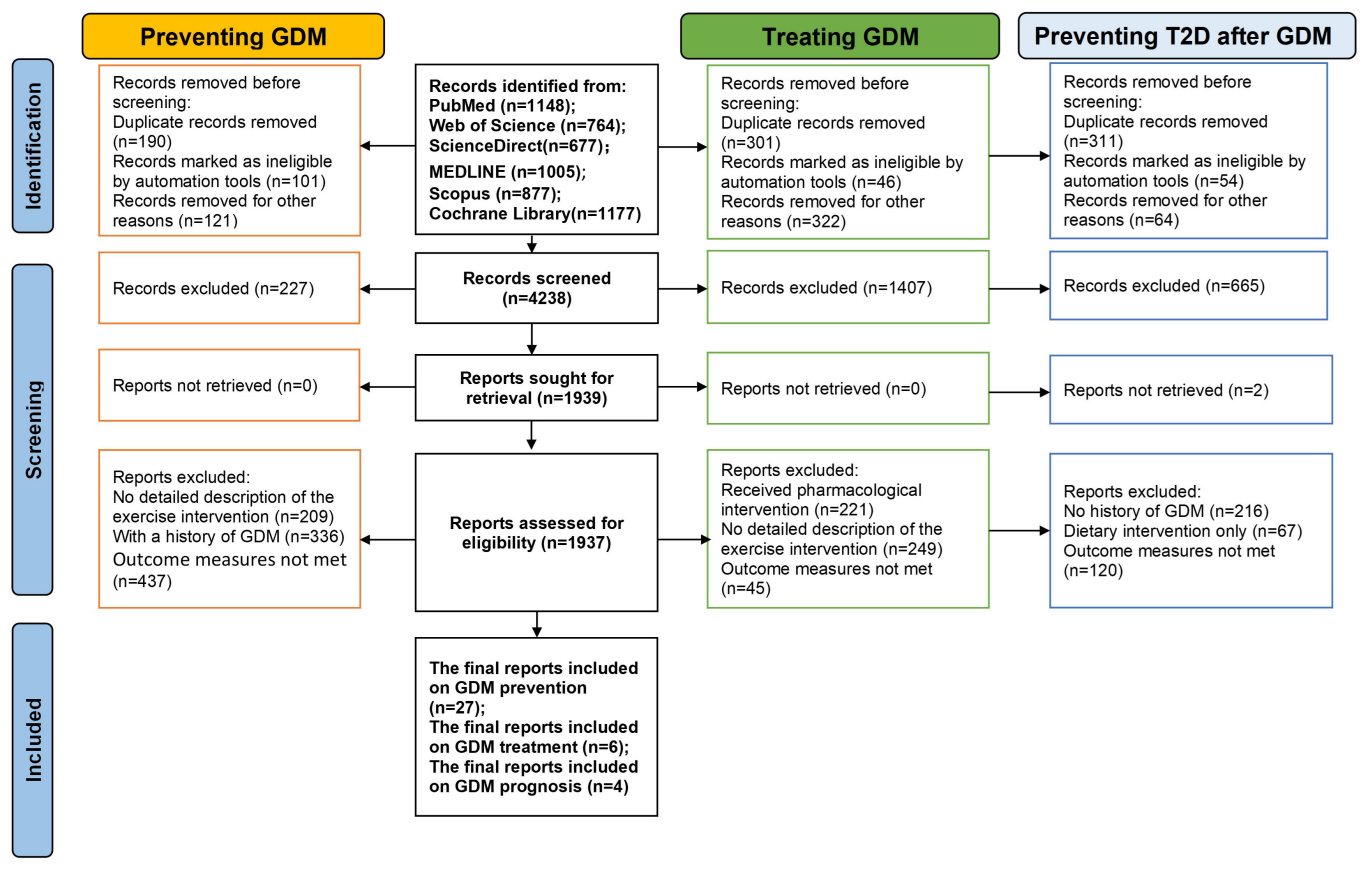
PRISMA flow diagram of study selection.

The final inclusion comprises 37 studies (**Figure 2**), with a cumulative sample size of 10,699 participants, the publication years ranged from 1999 to 2022. Among them, there are 27 reports specifically focusing on the relationship between exercise and the prevention of Gestational Diabetes Mellitus (GDM) (11–37), 6 reports on exercise and GDM treatment (38–43), and 4 reports on exercise and the prevention of T2D following GDM (44–47). Of course, in the post-GDM prognosis stage, other chronic diseases may also emerge, but this study specifically selected the highest-risk T2D for investigation.

### 3.2 Characteristics of the included studies

In the end, we included basic information from 37 studies, as detailed in **Table 1**. The table includes sample information for both the experimental and control groups, covering the country or region of the sample, sample size, lost samples, subjects’ age, body mass index (BMI), exercise intervention duration, exercise attendance rate, perceived exercise intensity, and outcome indicators. The exercise intervention plans and implementation details for the experimental group can be found in **Table 1**, which includes comprehensive exercise prescriptions for all studies. By summarizing and integrating the exercise interventions for GDM and subsequent T2D, along with their basic information, we arrived at the following conclusions. Firstly, compared to the post-GDM stage, exercise prescriptions for T2D prevention in the pre-GDM and treatment stages are more precise and detailed. Ninety percent of the studies provided detailed descriptions of the frequency, intensity, time, type, volume, and progression monitoring (FITT-VP) principles, offering a comprehensive implementation process for the exercise prescription (**Supplementary File 2**). Secondly, the exercise intervention period in the pre-GDM and treatment stages is relatively short, typically ranging from 6 to 30 weeks of intervention during pregnancy, whereas the exercise intervention time in the post-GDM stage is usually over one year (**Table 1**). Thirdly, the average exercise adherence is higher in the pre-GDM and treatment stages (≥80%), while in contrast, exercise adherence in the post-GDM stage is generally lower (<50%) (**Table 1**). These factors may contribute to significant differences in intervention effects across different stages of GDM management. We first integrated relevant data from all studies and then used the results of meta-analyses to determine whether there were significant differences in the intervention effects of exercise management for GDM across these three stages.

**Table 1:** Information of included studies.

Author (Year)	Country	Management stages of GDM	Sample size (E/C)	Lost to follow-up (E/C)	Age	Baseline BMI	Intervention duration (week of pregnancy)	Borg’s scale	Exercise adherence	Outcome
Antoun 2020 (11)	England	GDM prevention	294/263	0/0	≥16	≥30	8th~39th	NA	NA	GDM incidence
Barakat 2012 (12)	Spain	GDM prevention	40/43	0/0	32 ± 4	22.7 ± 2.8	6th~39th	NA	high	GDM incidence
Barakat 2013 (13)	Spain	GDM prevention	225/225	15/7	31 ± 3	24.1 ± 4.1	10th~39th	10~12	high	GDM incidence
Barakat 2014 (14)	Spain	GDM prevention	107/93	0/3	31.54 ± 3.86	23.78 ± 4.4	9th~40th	12~13	NA	GDM incidence
Barakat 2019 (8)	Spain	GDM prevention	260/260	26/38	31.75 ± 4.68	23.50 ± 3.79	8th~39th	12~14	high	GDM incidence
Bisson 2015 (16)	Canadian	GDM prevention	25/25	1/1	30.5 ± 3.7	35.2 ± 5.4	15th~27th	NA	moderate	GDM incidence
Callaway 2010 (17)	Australia	GDM prevention	25/25	3/6	NA	NA	12th~36th	NA	moderate	GDM incidence
Cordero 2015 (18)	Spain	GDM prevention	101/156	0/0	33.24 ± 4.3	22.5 ± 3.2	10 th~40th	12~14	high	GDM incidence
Ko 2014 (22)	American	GDM prevention	591/605	13/59	26.0 ± 4.8	26.0 ± 4.8	18th~36th	NA	low	GDM incidence
da Silva 2017 (19)	American	GDM prevention	213/426	8/19	27.2 ± 5.3	25.2 ± 4.1	16th~36th	12~14	NA	GDM incidence
Elden 2008 (20)	Sweden	GDM prevention	131/130	1/1	29.8 ± 4.2	NA	6 weeks	NA	high	GDM incidence
Garnæs 2016 (21)	England	GDM prevention	50/50	9/14	33.1 ± 6 5.2	34.3 ± 5.6	20th~37th	NA	NA	GDM incidence
Kong 2014 (23)	American	GDM prevention	19/23	1/4	28.6 ± 5.3	34.7 ± 4.6	12th~35 h	NA	NA	GDM incidence
Nobles 2015 (24)	American	GDM prevention	143/147	19/20	16~40	25~40	12 weeks	NA	high	GDM incidence
Okido 2015 (25)	Brazil	GDM prevention	48/48	22/14	23.41 ± 5.31	24.12 ± 4.31	20th~36th	NA	NA	GDM incidence
Oostdam 2012(26)	Netherlands	GDM prevention	62/59	13/7	30.8 ± 5.2	33.0 ± 3.7	24th~40th	12~14	low	GDM incidence
Pelaez 2019 (27)	Spain	GDM prevention	100/201	0/0	31.07 ± 3.19	24.1 ± 4.4	12th~36th	12~14	high	GDM incidence
Price 2012(28)	American	GDM prevention	31/31	0/0	30.5 ± 5	26.6 ± 3.1	12th~36th	12~14	high	GDM incidence
Renault 2014-PA(29)	Denmark	GDM prevention	125/134	0/0	30.9 ± 4.9	34.1 ± 4.4	13th~37th	NA	high	GDM incidence
Ruiz 2013 (30)	Spain	GDM prevention	481/481	0/0	31.6 ± 4	23.7 ± 3.9	9th~39th	10~12	high	GDM incidence
Seneviratne 2015 (31)	New Zealand	GDM prevention	38/37	1/0	18~40	≥25	20th~35th	NA	low	GDM incidence
Simmons 2015 (32)	9 European countries	GDM prevention	50/50	15/14	33.1 ± 5.2	34.5 ± 4.5	20th ~37th	NA	NA	GDM incidence
Simmons 2017 (33)	9 European countries	GDM prevention	110/105	21/11	31.7 ± 5.1	33.7 ± 4.0	24th~37th	NA	NA	GDM incidence
Tomić V 2013 (34)	Croatia	GDM prevention	166/168	0/0	28.9	23.1 ± 4.1	6th~40th	NA	NA	GDM incidence
Uria-M 2022 (35)	Spain	GDM prevention	130/130	28/29	33.80 ± 3.27	22.70 ± 4.17	8th~39th	12~14	high	GDM incidence
Ussher 2015 (36)	England	GDM prevention	392/393	2/2	27.2 ± 6.1	25.6 ± 5.0	15th~40th	NA	low	GDM incidence
Wang 2016 (37)	China	GDM prevention	150/150	18/17	32.14 ± 4.57	26.75 ± 2.74	12th~37th	12~14	high	GDM incidence
Zhao 2022 (43)	China	GDM treatment	64/60	0/0	31.2 ± 4.1	NA	13 weeks	13~14	moderate	75g-OGTT
Jin 2022 (40)	China	GDM treatment	67/67	2/1	33.51 ± 4.27	21.31 ± 3.20	12 weeks	NA	high	75g-OGTT
Gao 2019 (38)	China	GDM treatment	49/50	6/4	31.84 ± 5.19	23.03 ± 5.22	6 weeks	13	high	75g-OGTT
Kokic 2018 (41)	Croatia	GDM treatment	20/22	2/2	32.78 ± 3.83	24.39 ± 4.89	6 weeks	NA	high	75g-OGTT
Halse 2015 (39)	Australia	GDM treatment	20/20	0/0	34 ± 5	25.2 ± 6.7	26th~37th	NA	high	75g-OGTT
Wu 2022 (42)	China	GDM treatment	75/75	NA	28.75 ± 3.93	22.00 ± 1.94	4 weeks	NA	high	75g-OGTT
Wein 1999 (47)	Melbourne	GDM prognosis	100/100	3/4	39.5	25.2	6 years	NA	low	T2D incidence
Tandon 2022 (46)	India et.al	GDM prognosis	800/801	0/0	30.9 ± 4.9	26.6 ± 4.6	12 months	NA	low	T2D incidence
Rather 2008 (45)	American	GDM prognosis	117/122	3/4	43.0 ± 7.6	34.2 ± 6.2	3 years	NA	low	T2D incidence
Cheung 2011 (44)	Sydney	GDM prognosis	22/21	4/5	37	27.8	12 months	NA	moderate	T2D incidence

The high, moderate, and low levels of adherence with exercise refer to attendance rates equal to or greater than 80%, between 50% and 80%, and less than or equal to 50%, respectively. “NA” indicates that information is not available in this context. “Borg’s scale” refers to the “Borg Rating of Perceived Exertion (RPE) scale,” which is commonly used to subjectively assess exercise intensity. It allows individuals to rate their perceived exertion during physical activity, helping to adjust and monitor the intensity level of the exercise (6: No exertion, 7-8: Extremely light; 9-10: Very light, 11-12: Light, 13-14: Somewhat hard, 15-16: Hard, 17-18: Very hard, 19-20: Maximal exertion).

### 3.3 Quality assessment of included studies

According to the preliminary risk assessment for publication bias as recommended by the Cochrane Collaboration. The overall risk of bias in the 37 included studies were judged to have a low risk of bias (**Figure 1**). Among them, the highest risk is “blinding of participants and personnel” because it is challenging to adhere to this measure in exercise therapy (48).

### 3.4 Publication bias analysis

**Figure 3:**
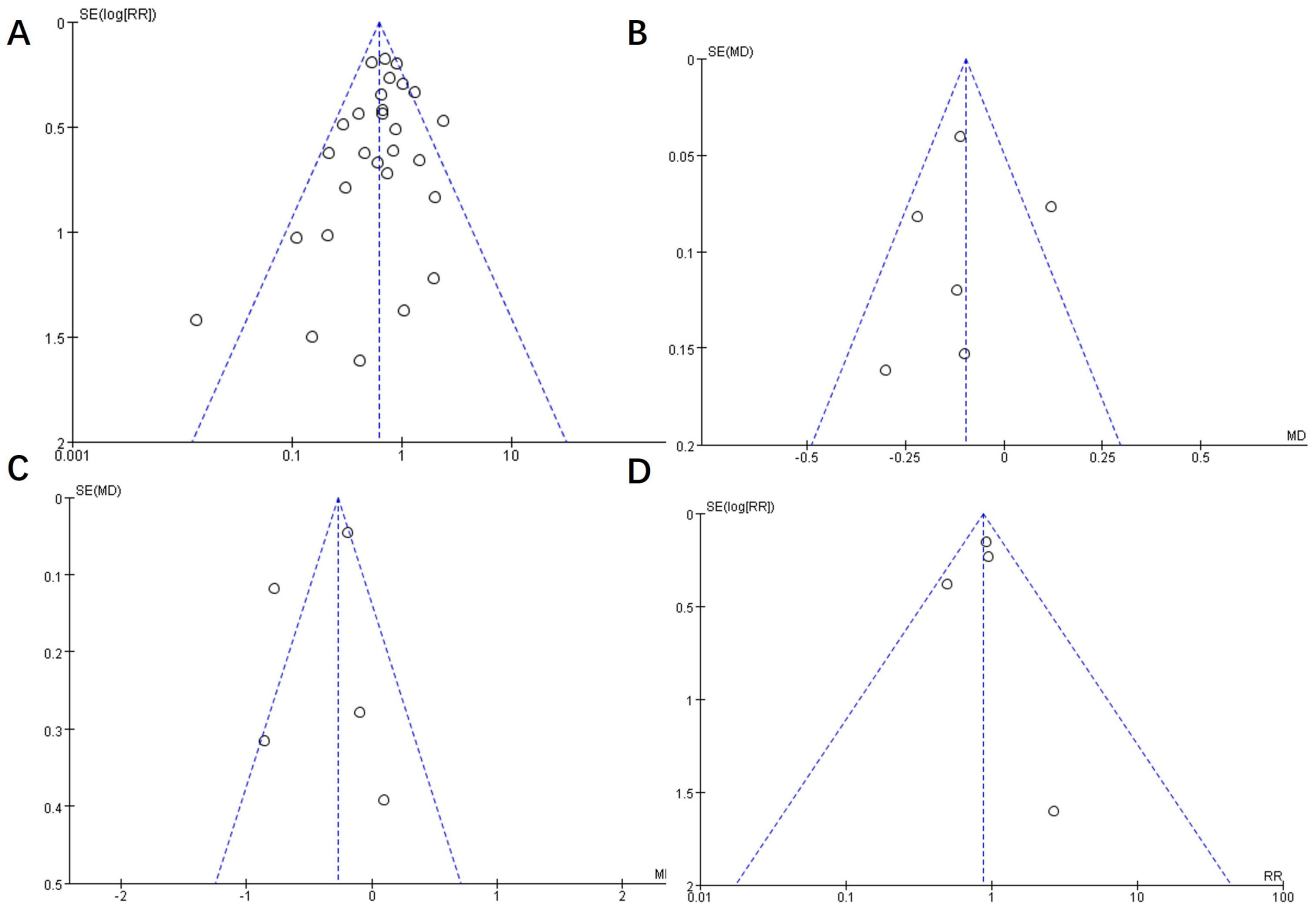
Funnel plot of publication bias of included studies. **(A)** displays a funnel plot of incidence data for exercise therapy in the prevention of GDM, while **(B, C)** respectively present funnel plots of fasting blood glucose and blood glucose two hours after a 75g -OGTT in the treatment of GDM. **(D)** shows a funnel plot of incidence data for exercise therapy in the prevention of progression from GDM to T2D.

The symmetrical shape of the funnel plot displayed in **Figure 3** suggested that the risk of publication bias was low. Due to the limited inclusion of studies in **Figures 3B**–**D** (n < 10), the results primarily rely on observations from **Figure 3A**.

### 3.5 Outcomes of meta-analysis

#### 3.5.1 The overall effect of exercise prescription intervention for GDM prevention

**Figure 4:**
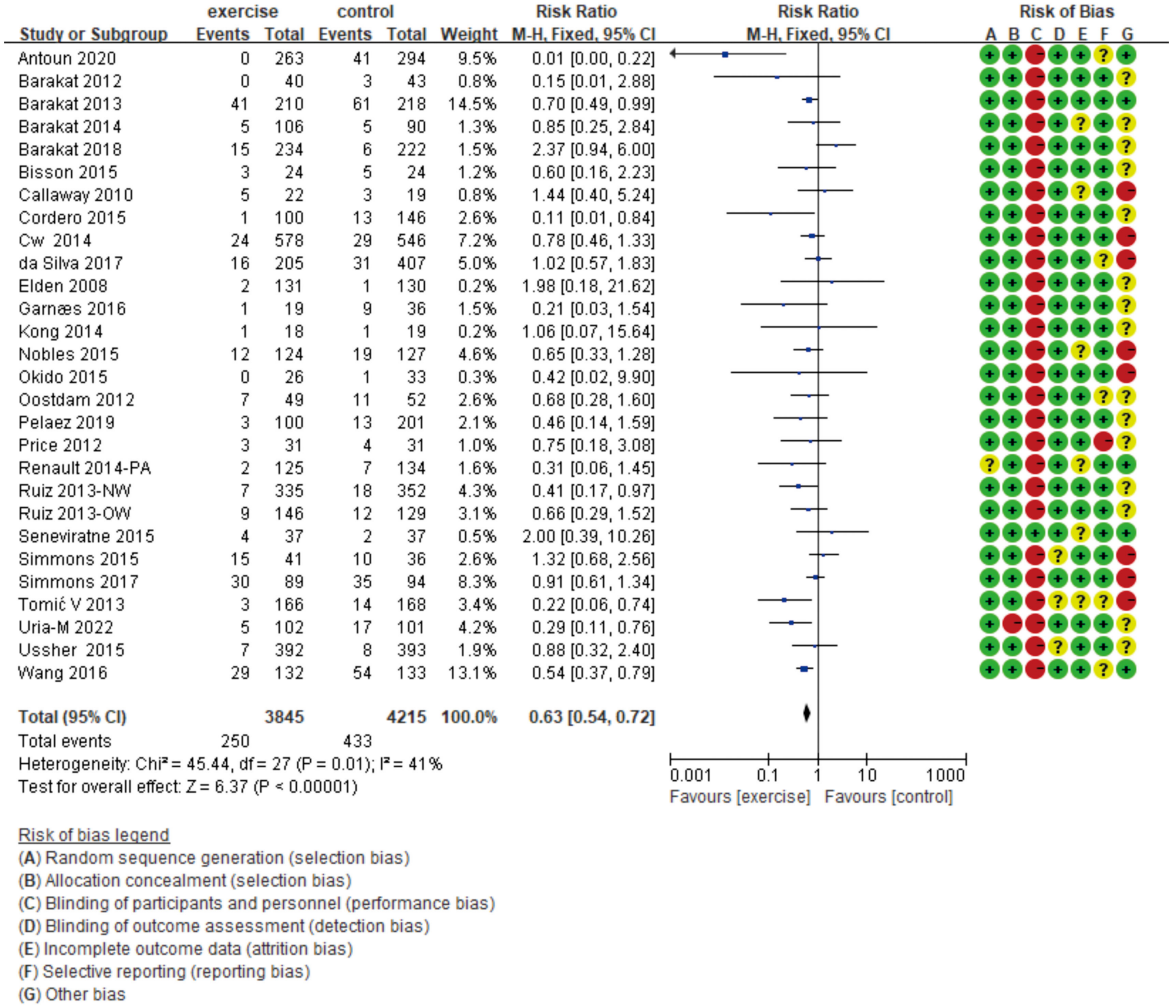
The forest plot illustrating the overall effect of exercise prescription intervention for GDM prevention.

As shown in **Figure 4**, in this meta-analysis focused on exercise prescription for the prevention of GDM, we included 27 randomized controlled trials (RCTs) (11–37) that compared the incidence of GDM between the control group and the exercise group. The results indicate that the exercise group had a lower GDM incidence compared to the control group (*p*<0.00001), with a relative risk (RR) of 0.63 and a 95% confidence interval (95%CI) ranging from 0.54 to 0.72, suggesting a significant reduction in GDM incidence in the exercise group. Furthermore, the heterogeneity statistic I^2^ was 41%, which is less than 50%, indicating relatively minor variability among the included studies.

#### 3.5.2 The therapeutic effect of exercise prescription intervention on GDM

**Figure 5:**
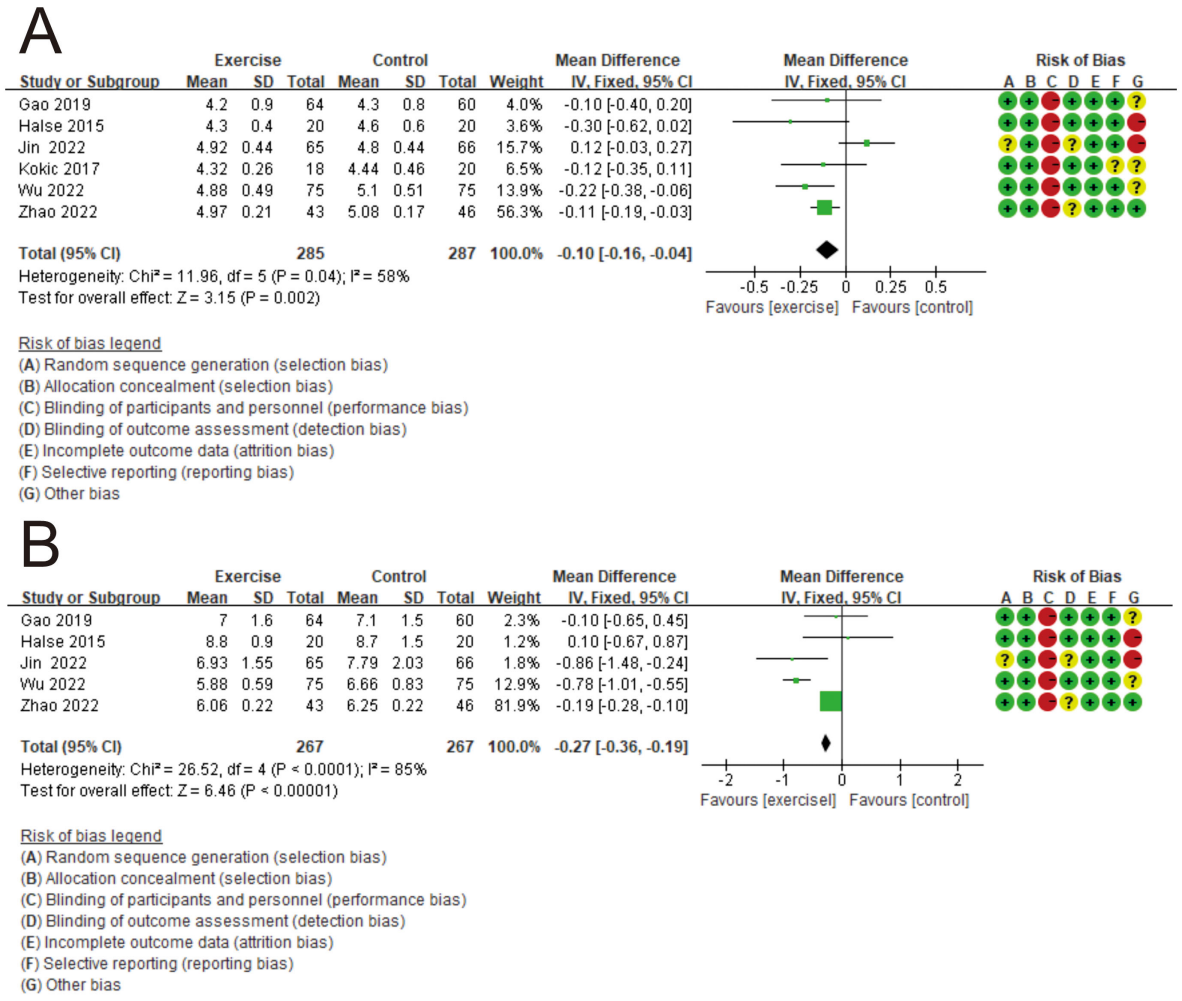
This forest plot illustrates the beneficial effects of exercise prescription intervention on 75g-OGTT. **(A)** The forest plot illustrating the control effect of exercise prescription intervention on fasting blood glucose levels. **(B)** The forest plot illustrating the control effect of exercise prescription intervention on 2-h postprandial blood glucose levels.

We incorporated data from 6 six RCTs (38–43). **Figure 5** depict the influence of exercise therapy on fasting blood glucose and blood glucose levels two hours after a 75g-OGTT in the exercise group compared to the control group. **Figure 5A** illustrates the fasting blood glucose results for both groups after the intervention, with a mean difference (MD) of -0.10 and a 95% CI of (-0.16, -0.04), along with an I² statistic of 58%. **Figure 5B** depicts the results of blood glucose levels two hours after a 75g-OGTT for both groups post-intervention, with an MD of -0.27 and a 95% CI of (-0.36, -0.19), and an I² of 85%. In both cases, the 95% confidence intervals do not include zero, indicating that these differences in effect are statistically significant. The overall results from both forest plots are skewed in favor of the exercise group, suggesting that exercise therapy is more effective in controlling blood glucose levels compared to the control group. The I² values of 58% and 85% for the meta-analysis results of fasting blood glucose and blood glucose levels two hours after the test respectively indicate a moderate and high level of heterogeneity.

#### 3.5.3 The effectiveness of exercise in preventing the development of T2D after GDM diagnosis.

**Figure 6:**
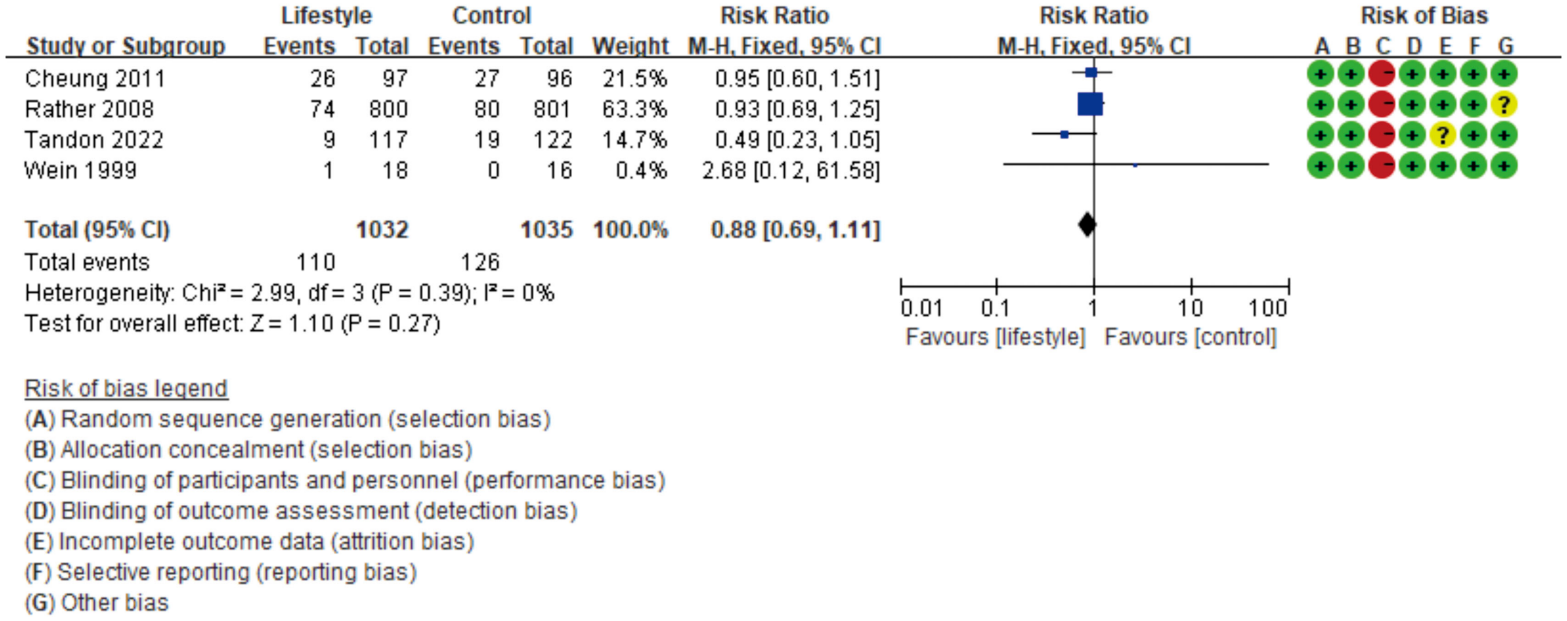
The forest plot illustrating the effectiveness of lifestyle intervention in preventing type 2 diabetes after GDM.

Lifestyle intervention, encompassing dietary control and physical exercise, stands as a primary method for treating T2D. For women with a history of GDM, there exists the potential for T2D development postpartum. However, the need for extended follow-up introduces the challenge of potential sample loss. We identified four RCTs (44–47) that provided data on T2D incidence rates in both lifestyle intervention and control groups. As indicated in **Figure 6**, the results reveal a Relative Risk (RR) of 0.88, a 95% CI spanning from 0.69 to 1.11, and an I² statistic of 0%. These findings suggest that in these studies, lifestyle intervention did not significantly affect the risk of women with a history of GDM developing T2D.

## 4 Discussion

After a comprehensive study of the meta-analysis of exercise therapy in the prevention, treatment, and prognosis of GDM, this study concludes that exercise therapy shows significant intervention effects in preventing and treating GDM (**Figures 4**, **5**), while its effectiveness in preventing T2D after GDM is not significant (**Figure 6**). We conducted in-depth analyses for these two distinct outcomes and proposed recommendations for exercise therapy in GDM management.

In GDM prevention and treatment studies, the “FITT-VP” framework was utilized for quantitative control (**Supplementary File 2**), ensuring the safety of exercise during pregnancy and experimental attendance rates. However, in studies focusing on preventing T2D after GDM, lifestyle interventions typically provided only verbal advice, lacking quantitative exercise prescriptions. According to Choi et al.’s research (2013) (49), quantitative exercise prescriptions can enhance patients’ adherence to exercise. Therefore, a focus on quantitative exercise regimens is crucial in lifestyle interventions during the post-GDM period.

As women transition into the postpartum period, home-based exercise becomes part of their daily routine, and face-to-face supervision frequency decreases as their contact with the healthcare system diminishes (**Supplementary File 2**). This makes the implementation of postpartum exercise regimens more complex. Even with established quantitative exercise plans, women with a history of GDM still demonstrate lower actual adherence to exercise in preventing T2D postpartum (**Supplementary File 2**). Additionally, challenges related to exercise adherence, long-term supervision, GDM post-treatment education, and follow-up contribute to the difficulty of sustaining postpartum exercise. Therefore, overcoming challenges related to exercise adherence, long-term supervision, GDM post-treatment education, and follow-up is crucial to enhancing the intervention effects of exercise therapy in preventing T2D after GDM.

Considering the challenges in implementing exercise therapy postpartum, studies indicate that pregnancy is a critical period for preventing type 2 diabetes since women have frequent contact with the healthcare system during this time, allowing for the shaping of postpartum lifestyle choices (50, 51). Thus, this study suggests integrating the prevention of T2D in post-GDM patients with the prevention and treatment of GDM during pregnancy through exercise therapy. The aim is to leverage the favorable conditions of frequent contact between pregnant women and the healthcare system to help women acquire exercise skills, establish awareness of independent exercise, and lay the foundation for autonomous exercise postpartum.

**Figure 7:**
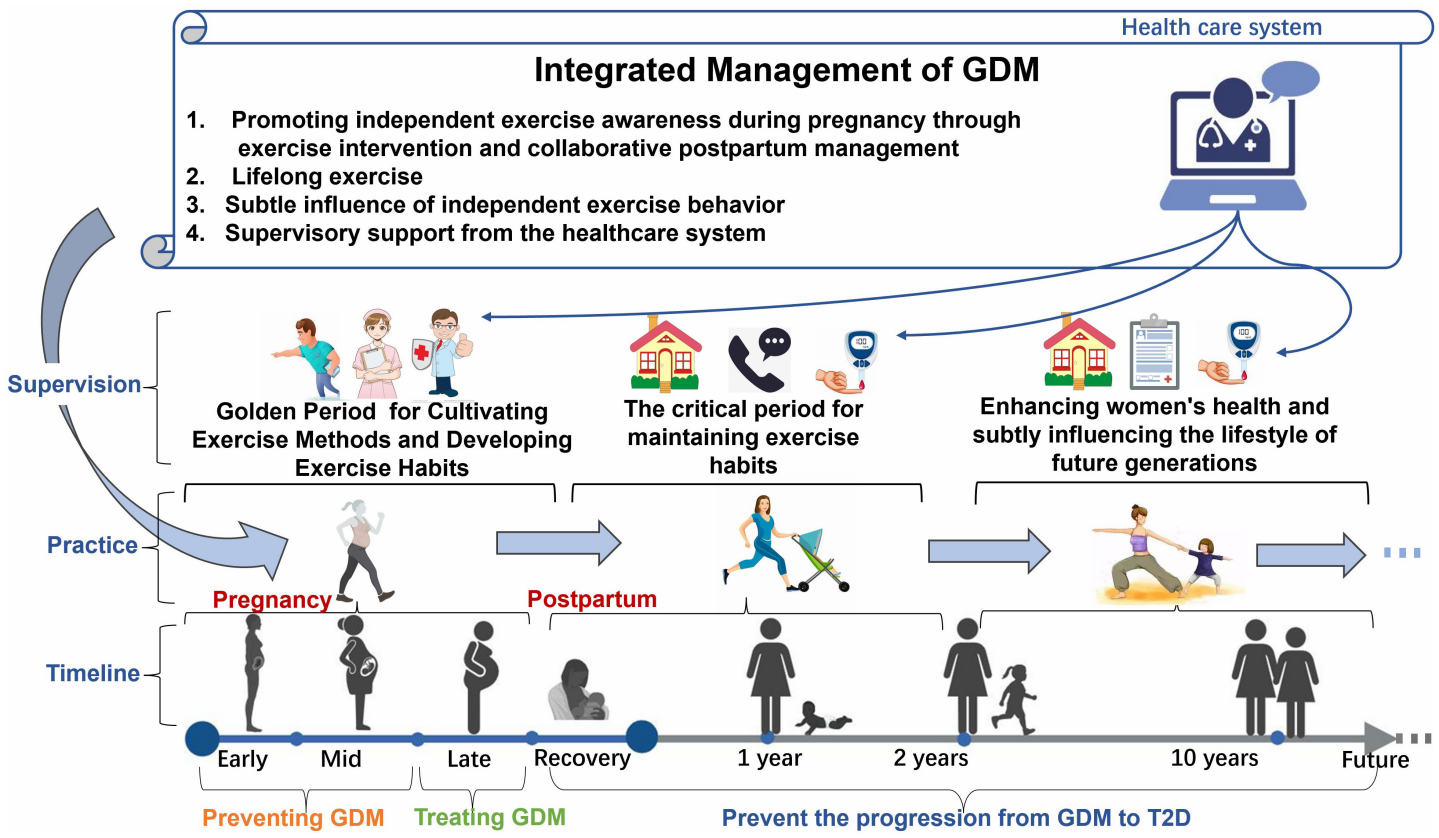
Comprehensive management model of exercise therapy for GDM.

Specifically, as shown in **Figure 7**, the management model includes several key aspects:

1 Pregnancy Exercise Intervention (GDM Prevention and Treatment Phases):

- Encourage healthcare professionals to develop personalized exercise prescriptions for each GDM patient, considering factors such as the patient’s health condition, exercise history, time constraints, and provide clear recommendations for exercise frequency, intensity, duration, and type.
- Provide face-to-face professional supervision, including obstetricians, fitness coaches, etc., to enhance pregnant women’s adherence to exercise. Professional supervision is crucial in helping pregnant women develop their awareness and methods of autonomous exercise and educating them about post-GDM sequelae.

2 Postpartum Exercise Intervention (Post-GDM Prognosis Phase):

- Within the first 1-2 years postpartum (52, 53): The personalized exercise prescriptions from the pregnancy period remain applicable, and women continue to maintain good exercise habits based on the exercise methods during pregnancy.
- Beyond 2 years (54): Employ innovative, flexible exercise plans tailored to individual needs and interests, such as diverse parent-child exercise programs.
- Emphasize personalized, long-term support using self-management education techniques, such as cognitive-behavioral techniques and motivational conversations, to help patients establish and maintain healthy exercise habits.

3 Lifelong Exercise and Implicit Autonomous Exercise Behavior:

- Continuously advocate and support women to maintain lifelong exercise, ensuring their active participation in physical activities through detailed exercise guidelines and ongoing training.
- Foster a positive family atmosphere, inspiring the next generation’s autonomous exercise behavior through leading by example. This creates a healthy habit of physical activity in the family, laying a solid foundation for the children’s health.

4 Supervisory Support from the Healthcare System:

- In the prevention and treatment stages of GDM, provide face-to-face supervision by a professional team as much as possible.
- In the post-GDM phase, create a support network where patients and healthcare professionals can share experiences, solve problems, and motivate each other. This can be achieved through online social platforms, regular meetings, etc. Additionally, strengthen telephone follow-ups within the first 1-2 years postpartum and encourage women to monitor blood glucose levels.

Through this integrated exercise therapy management model, the goal is to instill good exercise habits during pregnancy, perpetuate and consolidate these habits postpartum, ultimately forming a healthy pattern of lifelong exercise. By collaborating with patients and the healthcare system, providing comprehensive support and training, more significant effects can be achieved. In long-term health management, exercise not only improves the health of mothers but also has a subtle influence on shaping the next generation’s good habits of autonomous exercise. Such a family exercise atmosphere contributes to promoting health throughout the entire life cycle, conveying the importance of physical activity within the family and establishing a solid foundation for the health of the children.

### 4.1 Limitations

There are limited studies meeting the inclusion criteria (T2D incidence rates) on the effectiveness of exercise therapy in preventing T2D after GDM, which may introduce bias. Future research should focus on long-term follow-up to expand data on T2D incidence rates. Additionally, while our meta-analysis focused on the metabolic improvements from exercise therapy in women with GDM, it did not assess its effects on cardiac function, particularly echocardiographic parameters like myocardial strain. Since GDM women may experience subclinical myocardial dysfunction (55, 56). Therefore, exercise therapy may have potential beneficial effects on the myocardial strain parameters in these women. Future research should further explore this topic to assess the role of exercise therapy in preventing and treating GDM-related cardiac complications. This will provide more comprehensive evidence for the management of GDM.

## 5 Conclusions

This study highlights that while exercise therapy has short-term benefits in preventing and treating GDM, its effectiveness in preventing T2D post-GDM is limited by prolonged interventions and challenges in long-term follow-up. To address metabolic concerns and instill positive exercise habits in future generations, the manuscript proposes an integrated approach for comprehensive GDM management, aimed at promoting lifelong health. Additionally, as women with GDM may have subclinical myocardial dysfunction, further research should explore exercise therapy’s role in preventing GDM-related cardiac complications. Larger-scale, long-term studies are needed to confirm its effectiveness in preventing T2D and cardiac issues post-GDM, ensuring a more comprehensive management strategy.

## Data Availability

The original contributions presented in the study are included in the article/**Supplementary Material**. Further inquiries can be directed to the corresponding author.

## Supplementary material

ADDITIONAL FILE 1 Search Strategy.

ADDITIONAL FILE 2 The exercise prescription and implementation strategies.

ADDITIONAL FILE 3 Outcome measurement.

ADDITIONAL FILE 4 Risk of Bias.

ADDITIONAL FILE 5 Abbreviations.

ADDITIONAL FILE 6 Review Protocol.

PRESENTATION 1 PRISMA Checklist.

## References

[1] Wang H Li N Chivese T Werfalli M Sun H Yuen L . IDF diabetes atlas: estimation of global and regional gestational diabetes mellitus prevalence for 2021 by international association of diabetes in pregnancy study group's criteria. Diabetes Res Clin Practice. (2022) 183:109050. doi: 10.1016/j.diabres.2021.109050 34883186

[2] Moses RG . The recurrence rate of gestational diabetes in subsequent pregnancies. Diabetes Care. (1996) 19:1348–50. doi: 10.2337/diacare.19.12.1348 8941462

[3] Chiefari E Arcidiacono B Foti D Brunetti A . Gestational diabetes mellitus: an updated overview. J Endocrinological Invest. (2017) 40:899–909. doi: 10.1007/s40618-016-0607-5 28283913

[4] Vounzoulaki E Khunti K Abner SC Tan BK Davies MJ Gillies CL . Progression to type 2 diabetes in women with a known history of gestational diabetes: systematic review and meta-analysis. Bmj. (2020) 369:m1361. doi: 10.1136/bmj.m1361 32404325 PMC7218708

[5] Diabetes Prevention Program Research G . 10-year follow-up of diabetes incidence and weight loss in the Diabetes Prevention Program Outcomes Study. Lancet. (2009) 374:1677–86. doi: 10.1016/S0140-6736(09)61457-4 PMC3135022 19878986

[6] Davenport MH Kathol AJ Mottola MF Skow RJ Meah VL Poitras VJ . Prenatal exercise is not associated with fetal mortality: a systematic review and meta-analysis. Br J Sports Med. (2019) 53:108–15. doi: 10.1136/bjsports-2018-099773 30337346

[7] Smith BJ Cheung NW Bauman AE Zehle K McLean M . Postpartum physical activity and related psychosocial factors among women with recent gestational diabetes mellitus. Diabetes Care. (2005) 28:2650–4. doi: 10.2337/diacare.28.11.2650 16249534

[8] Buelo AK Kirk A Lindsay RS Jepson RG . Exploring the effectiveness of physical activity interventions in women with previous gestational diabetes: A systematic review of quantitative and qualitative studies. Prev Med Rep. (2019) 14:100877. doi: 10.1016/j.pmedr.2019.100877 31110933 PMC6510702

[9] Page M McKenzie J Bossuyt P Boutron I Hoffmann T Mulrow C . The PRISMA 2020 statement: an updated guideline for reporting systematic reviews. BMJ (Clinical Res ed). (2021) 372:n71. doi: 10.1136/bmj.n71 PMC8005924 33782057

[10] Cumpston MS McKenzie JE Welch VA Brennan SE . Strengthening systematic reviews in public health: guidance in the Cochrane Handbook for Systematic Reviews of Interventions, 2nd edition. J Public Health (Oxf). (2022) 44:e588–92. doi: 10.1093/pubmed/fdac036 PMC9715291 35352103

[11] Antoun E Kitaba NT Titcombe P Dalrymple KV Garratt ES Barton SJ . Maternal dysglycaemia, changes in the infant's epigenome modified with a diet and physical activity intervention in pregnancy: Secondary analysis of a randomised control trial. PloS Med. (2020) 17:e1003229. doi: 10.1371/journal.pmed.1003229 33151971 PMC7643947

[12] Barakat R Cordero Y Coteron J Luaces M Montejo R . Exercise during pregnancy improves maternal glucose screen at 24-28 weeks: a randomised controlled trial. Br J Sports Med. (2012) 46:656–61. doi: 10.1136/bjsports-2011-090009 21948120

[13] Barakat R Pelaez M Lopez C Lucia A Ruiz JR . Exercise during pregnancy and gestational diabetes-related adverse effects: a randomised controlled trial. Br J Sports Med. (2013) 47:630–6. doi: 10.1136/bjsports-2012-091788 23365418

[14] Barakat R Perales M Bacchi M Coteron J Refoyo I . A program of exercise throughout pregnancy. Is it safe to mother and newborn? Am J Health Promotion. (2014) 29:2–8. doi: 10.4278/ajhp.130131-QUAN-56 24200335

[15] Barakat R Refoyo I Coteron J Franco E . Exercise during pregnancy has a preventative effect on excessive maternal weight gain and gestational diabetes. A randomized Controlled trial Braz J Phys Ther. (2019) 23:148–55. doi: 10.1016/j.bjpt.2018.11.005 PMC6428908 30470666

[16] Bisson M Alméras N Dufresne S Robitaille J Rhéaume C Bujold E . A 12-week exercise program for pregnant women with obesity to improve physical activity levels: an open randomised preliminary study. PloS One. (2015) 10:e0137742. doi: 10.1371/journal.pone.0137742 26375471 PMC4573757

[17] Callaway LK Colditz PB Byrne NM Lingwood BE Rowlands IJ Foxcroft K . Prevention of Gestational Diabetes Feasibility issues for an exercise intervention in obese pregnant women. Diabetes Care. (2010) 33:1457–9. doi: 10.2337/dc09-2336 PMC2890340 20357374

[18] Cordero Y Mottola MF Vargas J Blanco M Barakat R . Exercise is associated with a reduction in gestational diabetes mellitus. Med Sci Sports Exercise. (2015) 47:1328–33. doi: 10.1249/mss.0000000000000547 25333246

[19] da Silva SG Hallal PC Domingues MR Bertoldi AD da Silveira MF Bassani D . A randomized controlled trial of exercise during pregnancy on maternal and neonatal outcomes: results from the PAMELA study. Int J Behav Nutr Phys Activity. (2017) 14:1–11. doi: 10.1186/s12966-017-0632-6 PMC5741924 29273044

[20] Elden H Ostgaard H Fagevik-Olsen M Ladfors L Hagberg H . Treatments of pelvic girdle pain in pregnant women: adverse effects of standard treatment, acupuncture and stabilising exercises on the pregnancy, mother, delivery and the fetus/neonate. BMC complementary Altern Med. (2008) 8:34. doi: 10.1186/1472-6882-8-34 PMC2467402 18582370

[21] Garnæs K Mørkved S Salvesen Ø Moholdt T . Exercise training and weight gain in obese pregnant women: A randomized controlled trial (ETIP trial). PloS Med. (2016) 13:e1002079. doi: 10.1371/journal.pmed.1002079 27459375 PMC4961392

[22] Ko C Napolitano P Lee S Schulte S Ciol M Beresford S . Physical activity, maternal metabolic measures, and the incidence of gallbladder sludge or stones during pregnancy: a randomized trial. Am J perinatology. (2014) 31:39–48. doi: 10.1055/s-0033-1334455 23456902

[23] Kong K Campbell C Foster R Peterson A Lanningham-Foster L . A pilot walking program promotes moderate-intensity physical activity during pregnancy. Med Sci sports exercise. (2014) 46:462–71. doi: 10.1249/mss.0000000000000141 24002348

[24] Nobles C Marcus BH Stanek EJ Braun B Whitcomb BW Solomon CG . Effect of an exercise intervention on gestational diabetes mellitus A randomized controlled trial. Obstetrics Gynecology. (2015) 125:1195–204. doi: 10.1097/aog.0000000000000738 PMC4418021 25932848

[25] Okido M Valeri F Martins W Ferreira C Duarte G Cavalli R . Assessment of foetal wellbeing in pregnant women subjected to pelvic floor muscle training: a controlled randomised study. Int urogynecology J. (2015) 26:1475–81. doi: 10.1007/s00192-015-2719-4 26294205

[26] Oostdam N van Poppel MNM Wouters M Eekhoff EMW Bekedam DJ Kuchenbecker WKH . No effect of the FitFor2 exercise programme on blood glucose, insulin sensitivity, and birthweight in pregnant women who were overweight and at risk for gestational diabetes: results of a randomised controlled trial. Bjog-an Int J Obstetrics Gynaecology. (2012) 119:1098–107. doi: 10.1111/j.1471-0528.2012.03366.x 22616913

[27] Pelaez M Gonzalez-Cerron S Montejo R Barakat R . Protective effect of exercise in pregnant women including those who exceed weight gain recommendations: A randomized controlled trial. Mayo Clinic Proc. (2019) 94:1951–9. doi: 10.1016/j.mayocp.2019.01.050 31585579

[28] Price B Amini S Kappeler K . Exercise in pregnancy: effect on fitness and obstetric outcomes-a randomized trial. Med Sci sports exercise. (2012) 44:2263–9. doi: 10.1249/MSS.0b013e318267ad67 22843114

[29] Renault K Nørgaard K Nilas L Carlsen E Cortes D Pryds O . The Treatment of Obese Pregnant Women (TOP) study: a randomized controlled trial of the effect of physical activity intervention assessed by pedometer with or without dietary intervention in obese pregnant women. Am J obstetrics gynecology. (2014) 210:134.e1–9. doi: 10.1016/j.ajog.2013.09.029 24060449

[30] Ruiz JR Perales M Pelaez M Lopez C Lucia A Barakat R . Supervised exercise-based intervention to prevent excessive gestational weight gain: A randomized controlled trial. Mayo Clinic Proc. (2013) 88:1388–97. doi: 10.1016/j.mayocp.2013.07.020 24290112

[31] Seneviratne SN Derraik JGB Jiang YN McCowan LME Gusso S Cutfield WS . The sex of the foetus affects maternal blood glucose concentrations in overweight and obese pregnant women. J Obstetrics Gynaecology. (2017) 37:667–9. doi: 10.1080/01443615.2016.1256970 28019134

[32] Simmons D . Prevention of gestational diabetes mellitus: Where are we now? Diabetes Obes Metab. (2015) 17:824–34. doi: 10.1111/dom.12495 25974384

[33] Simmons D Devlieger R van Assche A Jans G Galjaard S Corcoy R . Effect of physical activity and/or healthy eating on GDM risk: the DALI lifestyle study. J Clin Endocrinol Metab. (2017) 102:903–13. doi: 10.1210/jc.2016-3455 PMC5460688 27935767

[34] Tomić V Sporiš G Tomić J Milanović Z Zigmundovac-Klaić D Pantelić S . The effect of maternal exercise during pregnancy on abnormal fetal growth. Croatian Med J. (2013) 54:362–8. doi: 10.3325/cmj.2013.54.362 PMC3760660 23986277

[35] Uria-Minguito A Silva-Jose C Sanchez-Polan M Diaz-Blanco A Garcia-Benasach F Martinez VC . The effect of online supervised exercise throughout pregnancy on the prevention of gestational diabetes in healthy pregnant women during covid-19 pandemic: a randomized clinical trial. Int J Environ Res Public Health. (2022) 19:14104. doi: 10.3390/ijerph192114104 36360995 PMC9655632

[36] Ussher M Lewis S Aveyard P Manyonda I West R Lewis B . The London Exercise And Pregnant smokers (LEAP) trial: a randomised controlled trial of physical activity for smoking cessation in pregnancy with an economic evaluation. Health Technol Assess (Winchester England). (2015) 19:vii–xxiv, 1-135. doi: 10.3310/hta19840 PMC4781101 26491878

[37] Wang C Wei YM Zhang XM Zhang Y Xu QQ Su SP . Effect of regular exercise commenced in early pregnancy on the incidence of gestational diabetes mellitus in overweight and obese pregnant women: A randomized controlled trial. Diabetes Care. (2016) 39:E163–4. doi: 10.2337/dc16-1320 27660125

[38] Guo H Zhang Y Li P Zhou P Chen L Li S . Evaluating the effects of mobile health intervention on weight management, glycemic control and pregnancy outcomes in patients with gestational diabetes mellitus. J endocrinological Invest. (2019) 42:709–14. doi: 10.1007/s40618-018-0975-0 30406378

[39] Halse RE Wallman KE Dimmock JA Newnham JP Guelfi KJ . Home-based exercise improves fitness and exercise attitude and intention in women with GDM. Med Sci Sports Exerc. (2015) 47:1698–704. doi: 10.1249/mss.0000000000000587 25412300

[40] Jin Y Chen ZF Li JQ Zhang W Feng SW . Effects of the original Gymnastics for Pregnant Women program on glycaemic control and delivery outcomes in women with gestational diabetes mellitus: A randomized controlled trial. Int J Nurs Stud. (2022) 132:104271. doi: 10.1016/j.ijnurstu.2022.104271 35660387

[41] Kokic IS Ivanisevic M Biolo G Simunic B Kokic T Pisot R . Combination of a structured aerobic and resistance exercise improves glycaemic control in pregnant women diagnosed with gestational diabetes mellitus. A randomised Controlled trial Women Birth. (2018) 31:E232–8. doi: 10.1016/j.wombi.2017.10.004 29055674

[42] Wu Y Xu M Zheng G . Application of diversified and quantitative management model of exercise intervention in patients with gestational diabetes mellitus. J Matern Fetal Neonatal Med. (2022) 35:5001–7. doi: 10.1080/14767058.2021.1874340 33478302

[43] Zhao HF Xie YP Zhao MJ Huang HB Liu CH Huang FF . Effects of moderate-intensity resistance exercise on blood glucose and pregnancy outcome in patients with gestational diabetes mellitus: A randomized controlled trial. J Diabetes Its Complications. (2022) 36:108186. doi: 10.1016/j.jdiacomp.2022.108186 35379538

[44] Cheung NW Smith BJ van der Ploeg HP Cinnadaio N Bauman A . A pilot structured behavioural intervention trial to increase physical activity among women with recent gestational diabetes. Diabetes Res Clin Practice. (2011) 92:E27–9. doi: 10.1016/j.diabres.2011.01.013 21316788

[45] Ratner R Christophi C Metzger B Dabelea D Bennett P Pi-Sunyer X . Prevention of diabetes in women with a history of gestational diabetes: effects of metformin and lifestyle interventions. J Clin Endocrinol Metab. (2008) 93:4774–9. doi: 10.1210/jc.2008-0772 PMC2626441 18826999

[46] Tandon N Gupta Y Kapoor D Lakshmi JK Praveen D Bhattacharya A . Effects of a lifestyle intervention to prevent deterioration in glycemic status among south asian women with recent gestational diabetes A randomized clinical trial. JAMA Network Open. (2022) 5:e220773. doi: 10.1001/jamanetworkopen.2022.0773 35234881 PMC8892226

[47] Wein P Beischer N Harris C Permezel M . A trial of simple versus intensified dietary modification for prevention of progression to diabetes mellitus in women with impaired glucose tolerance. Aust New Z J Obstetrics Gynaecology. (1999) 39:162–6. doi: 10.1111/j.1479-828X.1999.tb03363.x 10755770

[48] Smart NA Waldron M Ismail H Giallauria F Vigorito C Cornelissen V . Validation of a new tool for the assessment of study quality and reporting in exercise training studies: TESTEX. Int J Evid Based Healthc. (2015) 13:9–18. doi: 10.1097/xeb.0000000000000020 25734864

[49] Choi J Fukuoka Y Lee JH . The effects of physical activity and physical activity plus diet interventions on body weight in overweight or obese women who are pregnant or in postpartum: a systematic review and meta-analysis of randomized controlled trials. Prev Med. (2013) 56:351–64. doi: 10.1016/j.ypmed.2013.02.021 PMC3670949 23480971

[50] Adam S McIntyre HD Tsoi KY Kapur A Ma RC Dias S . Pregnancy as an opportunity to prevent type 2 diabetes mellitus: FIGO Best Practice Advice. Int J Gynecology Obstetrics. (2023) 160:56–67. doi: 10.1002/ijgo.v160.S1 PMC10107137 36635082

[51] Phelan S . Pregnancy: a “teachable moment” for weight control and obesity prevention. Am J Obstetrics Gynecology. (2010) 202:135.e1–8. doi: 10.1016/j.ajog.2009.06.008 PMC2815033 19683692

[52] McIntyre HD Peacock A Miller YD Koh D Marshall AL . Pilot study of an individualised early postpartum intervention to increase physical activity in women with previous gestational diabetes. Int J Endocrinol. (2012) 2012:892019. doi: 10.1155/2012/892019 22548057 PMC3324899

[53] Metzger BE Bybee DE Freinkel N Phelps RL Radvany RM Vaisrub N . Gestational diabetes mellitus. Correlations between the phenotypic and genotypic characteristics of the mother and abnormal glucose tolerance during the first year postpartum. Diabetes. (1985) 34 Suppl 2:111–5. doi: 10.2337/diab.34.2.s111 3888736

[54] O'Sullivan JB . Establishing criteria for gestational diabetes. Diabetes Care. (1980) 3:437–9. doi: 10.2337/diacare.3.3.437 7389559

[55] Li W Li Z Liu W Zhao P Che G Wang X . Two-dimensional speckle tracking echocardiography in assessing the subclinical myocardial dysfunction in patients with gestational diabetes mellitus. Cardiovasc Ultrasound. (2022) 20:21. doi: 10.1186/s12947-022-00292-3 35941651 PMC9361647

[56] Sonaglioni A Barlocci E Adda G Esposito V Ferrulli A Nicolosi GL . The impact of short-term hyperglycemia and obesity on biventricular and biatrial myocardial function assessed by speckle tracking echocardiography in a population of women with gestational diabetes mellitus. Nutrition Metab Cardiovasc Diseases. (2022) 32:456–68. doi: 10.1016/j.numecd.2021.10.011 34893411

