# Supplementary material for: Comprehensive management of gestational diabetes mellitus: practical efficacy of exercise therapy and sustained intervention strategies
Source: Front Endocrinol (Lausanne). 2024 Oct 3;15:1347754. doi: 10.3389/fendo.2024.1347754 (PMC11484007; doi:10.3389/fendo.2024.1347754)
Supplement: ADDITIONAL FILE 1 — Search Strategy. [file DataSheet1.zip › Additional file 5.DOCX]

**Additional file 5: Abbreviation.**

**Table 1 List of abbreviations**

| **Abbreviation** | **Full Form** |
| --- | --- |
| GDM | Gestational Diabetes Mellitus |
| T2D | Type 2 Diabetes |
| MD | Mean Difference |
| RCT | Randomized Controlled Trial |
| M | Mean |
| SD | Standard Deviation |
| 95%CI | 95% Confidence Interval |
| RR | Relative Risk |
| 75g-OGTT | The 75-gram oral glucose tolerance test |
| FITT-VP | frequency, intensity, time, type, volume, progression |
| BMI | Body Mass Index |
