# Supplementary material for: Comprehensive management of gestational diabetes mellitus: practical efficacy of exercise therapy and sustained intervention strategies
Source: Front Endocrinol (Lausanne). 2024 Oct 3;15:1347754. doi: 10.3389/fendo.2024.1347754 (PMC11484007; doi:10.3389/fendo.2024.1347754)
Supplement: ADDITIONAL FILE 1 — Search Strategy. [file DataSheet1.zip › Additional file 3.DOCX]

**Additional file 3: Outcome measurement**

**Table 1 The GDM incidence and sample size data of the exercise intervention group compared to the control group after exercise intervention.**

| Study（year） | Event | Total | Event | Total |
| --- | --- | --- | --- | --- |
| Wang 2016 | 29 | 132 | 54 | 133 |
| Ussher 2015 | 7 | 392 | 8 | 393 |
| Uria-M 2022 | 5 | 102 | 17 | 101 |
| Tomić V 2013 | 3 | 166 | 14 | 168 |
| Simmons 2017 | 30 | 89 | 35 | 94 |
| Simmons 2015 | 15 | 41 | 10 | 36 |
| Seneviratne 2015 | 4 | 37 | 2 | 37 |
| Ruiz 2013-OW | 9 | 146 | 12 | 129 |
| Ruiz 2013-NW | 7 | 335 | 18 | 352 |
| Renault 2014-PA | 2 | 125 | 7 | 134 |
| Price 2012 | 3 | 31 | 4 | 31 |
| Pelaez 2019 | 3 | 100 | 13 | 201 |
| Oostdam 2012 | 7 | 49 | 11 | 52 |
| Okido 2015 | 0 | 26 | 1 | 33 |
| Nobles 2015 | 12 | 124 | 19 | 127 |
| Kong 2014 | 1 | 18 | 1 | 19 |
| Garnæs 2016 | 1 | 19 | 9 | 36 |
| Elden 2008 | 2 | 131 | 1 | 130 |
| Cw 2014 | 24 | 578 | 29 | 546 |
| Cordero 2015 | 1 | 100 | 13 | 146 |
| Callaway 2010 | 5 | 22 | 3 | 19 |
| Bisson 2015 | 3 | 24 | 5 | 24 |
| Barakat 2018 | 15 | 234 | 6 | 222 |
| Barakat 2014 | 5 | 106 | 5 | 90 |
| Barakat 2013 | 41 | 210 | 61 | 218 |
| Barakat 2012 | 0 | 40 | 3 | 43 |
| Antoun 2020 | 0 | 263 | 41 | 294 |
| da Silva 2017 | 16 | 205 | 31 | 407 |

**Table 2 The 75g-OGTT results and sample size data of the exercise intervention group compared to the control group after exercise intervention.**

| FBG: | M | SD | N | M | SD | N |
| --- | --- | --- | --- | --- | --- | --- |
| Zhao 2022 | 4.97 | 0.21 | 43 | 5.08 | 0.17 | 46 |
| Wu 2022 | 4.88 | 0.49 | 75 | 5.1 | 0.51 | 75 |
| Kokic 2017 | 4.32 | 0.26 | 18 | 4.44 | 0.46 | 20 |
| Jin 2022 | 4.92 | 0.44 | 65 | 4.8 | 0.44 | 66 |
| Halse 2015 | 4.3 | 0.4 | 20 | 4.6 | 0.6 | 20 |
| Gao 2019 | 4.2 | 0.9 | 64 | 4.3 | 0.8 | 60 |
| 2h-BG: |  |  |  |  |  |  |
| Zhao 2022 | 6.06 | 0.22 | 43 | 6.25 | 0.22 | 46 |
| Wu 2022 | 5.88 | 0.59 | 75 | 6.66 | 0.83 | 75 |
| Jin 2022 | 6.93 | 1.55 | 65 | 7.79 | 2.03 | 66 |
| Halse 2015 | 8.8 | 0.9 | 20 | 8.7 | 1.5 | 20 |
| Gao 2019 | 7 | 1.6 | 64 | 7.1 | 1.5 | 60 |

**Table 3 The number of individuals progressing from GDM to T2D and sample size data for the exercise intervention group compared to the control group after exercise intervention.**

| Study（year） | Event | Total | Event | Total |
| --- | --- | --- | --- | --- |
| Wein 1999 | 26 | 97 | 27 | 96 |
| Tandon 2022 | 74 | 800 | 80 | 801 |
| Rather 2008 | 9 | 117 | 19 | 122 |
| Cheung 2011 | 1 | 18 | 0 | 16 |
