# Supplementary material for: Comprehensive management of gestational diabetes mellitus: practical efficacy of exercise therapy and sustained intervention strategies
Source: Front Endocrinol (Lausanne). 2024 Oct 3;15:1347754. doi: 10.3389/fendo.2024.1347754 (PMC11484007; doi:10.3389/fendo.2024.1347754)
Supplement: ADDITIONAL FILE 1 — Search Strategy. [file DataSheet1.zip › Additional file 2.DOCX]

**Additional file 2: The exercise prescription and implementation strategies.**

**Table 1. The exercise prescription details for inclusion in the study were organized based on the “FITT-VP”principles. These details encompassed the frequency, intensity, time, type, volume, and progression of the prescribed exercise.**

| Author (Year) | Exercise Prescription （FITT-VP） |
| --- | --- |
| Antoun 2020 | Detailed description of "FITT-VP" components and an in-depth explanation of the exercise prescription implementation process. The exercise sessions were supervised by a qualified physical activity and sport science professional. The exercise program consisted of complementary and interrelated |
| Barakat 2012 | The physical conditioning program consisted of 35-45minute sessions conducted three times a week (on Monday, Wednesday, and Friday). It included two land-based aerobic sessions and one aquatic activity session. The program began at the start of pregnancy (weeks 6-9) and continued until the end of the third trimester (weeks 38-39). The original plan was for each participant to complete 85 training sessions if there were no preterm deliveries. Participant used heart rate (HR) monitors during training sessions to ensure that exercise intensity remained light to moderate. This was defined as maintaining a heart rate consistently below 70% of their age-predicted maximum HR value (calculated as 220 minus their age). |
| Barakat 2013 | Exercise interventions were conducted in waves, with 10-12 participants in each group. The exercise group trained three days a week for 50-55 minutes per session. Training started in the 10th-12th week of pregnancy and continued until the end of the third trimester. The plan included an average of 85 training sessions per participant unless preterm delivery occurred. The program covered aerobic exercises, muscle strength, and flexibility, meeting medical standards. Heart rate monitors were used to maintain moderate exercise intensity (below 70% of maximum heart rate). The Borg scale (10 to 12) assessed perceived exertion, ranging from "fairly light" to "somewhat hard." |
| Barakat 2014 | 5-minute warm-up consisting of walking and static stretching. 30 minutes of exercises targeting major muscle groups in the legs, buttocks, and abdomen, as well as balancing exercises for 10 minutes. A 10-minute pelvic floor muscle training. A 5-minute cool-down period. The exercise program emphasized light-to-moderate-intensity aerobic activity, aiming to achieve a 55% to 60% maximal heart rate. Individualized heart rate targets were calculated using the Karvonen formula based on trimester, physical condition, and age, with adjustments guided by Borg Scale ratings (targeting level 12 to 13, corresponding to moderate/somewhat hard exercise). Participants wore heart rate monitors to ensure exercise intensity remained light to moderate. All sessions were supervised by qualified fitness specialists working with groups of 10-12 participants, with the presence of an obstetrician, and accompanied by music. They were conducted in a well-lit, spacious room at the University Hospital of Fuenlabrada under favorable environmental conditions (altitude 600 m, temperature 19-21°C, humidity 50-60%). Adequate calorie and nutrient intake were ensured for each participant before exercise sessions to maximize safety and program effectiveness. |
| Barakat 2019 | Detailed description of "FITT-VP" components and an in-depth explanation of the exercise prescription implementation process. The exercise intervention included moderate aerobic exercise performed three days per week (50-55 minutes per session) for 8-10 weeks to 38-39 weeks gestation. |
| Bisson 2015 | In accordance with the American College of Sports Medicine Guidelines, the exercise prescription consisted of three one-hour sessions per week, totaling 36 sessions over 12 weeks. Each session encompassed a 5–10-minute warm-up on a stationary ergo cycle, a 15–30-minute treadmill walks, a 20-minute muscular workout, and a cool-down. The duration of cardiovascular training progressively increased from 15 minutes in the first week to 30 minutes by the end of the first month. The muscular workout involved dynamic exercises for both lower and upper limbs, employing body weight, small weights, exercise balls, and strength equipment. Participants began with 1 set of 10-15 repetitions per exercise, progressing to 2 sets of 15 repetitions, with intensity tailored to their tolerance. To maintain motivation, the muscular workout was adjusted every 4 weeks. Exercise intensity was self-monitored using heart rate monitors and the modified Borg Scale, with targets set at 70% of peak heart rate (measured during the fitness test) and/or a perceived exertion score of 3-5 out of 10. Participants recorded session duration and mean heart rate in their exercise logs, and on days without training, they were encouraged to stay as active as possible. |
| Callaway 2010 | The detailed content of "FITT-VP" was not described, and the process of implementing the exercise prescription was introduced. |
| Cordero 2015 | The physical activity program was conducted from weeks 10 to the end of the third trimester, comprising 50- to 60-minute sessions three times a week. Two sessions were land-based in a gym hall, and one was a water-based activity in small and large pool tanks. Exercise intensity was regulated using Borg's scale, aiming to maintain a level of 12-14, signifying somewhat strong effort. Maternal heart rate was monitored using Accrue Plus HR monitors, and exercise intensity was adjusted to stay below 60% of the calculated heart rate reserve [(220 - age) - (resting heart rate) * 60%] + resting heart rate. The sessions were supervised by a qualified fitness specialist, working with groups of 10-12 women, with the support of an obstetrician, to ensure patient safety, adherence, and program effectiveness. |
| Ko 2014 | The intervention involved a moderate to vigorous exercise program aimed at increasing participants' exercise frequency and duration. It began with 30-minute sessions three times per week and aimed to reach four to five sessions per week lasting 45 to 60 minutes each. Participants received structured supervised sessions and were also taught self-motivation and behavior change techniques during monthly meetings with the exercise interventionist. In the first month, the exercise frequency gradually increased to three gym sessions per week and one to two home sessions, each lasting 30 minutes. In months 2 through 6, participants continued with three gym sessions per week and one to two home sessions. The Women in the intervention program participated from enrollment until 36 weeks of gestation. They also received regular mailings at home to support their physical activity goals and behavior change. Participants' exercise goals could adapt as their exercise capacity changed over time. If any medical contraindications to exercise emerged, participants were withdrawn from the intervention but analyzed based on their original assignment on an intention-to-treat basis. |
| da Silva 2017 | The exercise training program started between 16 and20 weeks’ gestation and was continued for at least16 weeks. Women in the intervention group received a structured, individually supervised, moderate intensity exercise program for 1 hour 3 days/week planned according to the ACOG recommendations. Each session involved warm-up, aerobic activities (treadmill or stationary bike), strength training (dumbbells, machines or elastic bands), and stretching exercises. The exercise intensity was measured according to each woman’s perceived effort (within the range of 12 to 14 on the Borg Scale) [17]. A mean of 48 training sessions were planned for each participant. The training sessions were grouped into three stages. The first stage (week 1 to 4) began with 5 min warm-up period, 15 min aerobic exercise, 35 min strength training/floor exercises (sets: 3 × 12 repetitions), and 5 min stretching. The second stage (week 5 to 10) started with 5 min warm-up period, 20 min aerobic exercise, 30 min strength training/floor exercises (sets: 3 × 10 repetitions), and 5 min stretching. Lastly, the third stage (11 to 16) began with 5 min warm-up period, 25 min aerobic exercise, 25 min strength training/floor exercises (sets: 3 × 8 repetitions), and 5 min stretching. Sessions were guided by a team of five trained physical education professionals. In order to offer personalized supervision, each shift counted on the presence of two physical education professionals and a maximum of six pregnant women per hour. The intervention program was performed at Federal University of Pelotas at the gym of the Physical Education School. |
| Elden 2008 | Detailed description of "FITT-VP" components and an in-depth explanation of the exercise prescription implementation process. |
| Garnæs 2016 | Detailed description of "FITT-VP" components and an in-depth explanation of the exercise prescription implementation process. |
| Kong 2014 | The intervention in this study consisted of an unsupervised walking program. After an initial training session, participants were encouraged to follow the 2008 U.S. physical activity guidelines, aiming for a minimum of 150 minutes of moderate physical activity (PA) per week during pregnancy. They were advised to spread their walking throughout the week, aiming for 30 minutes of walking on at least 5 days a week. Treadmills were provided to intervention participants for home use, with a total of 16 treadmills provided. Two participants who had their own treadmills at home were exempt from receiving one. Participants were asked to log the location and duration of their walks but didn't report the intensity. The unsupervised walking program typically started between weeks 12 and 15 of pregnancy and continued until at least week 35. All intervention participants were able to complete at least 20 weeks, with the initial two weeks serving as an acclimation period where they gradually increased their walking time from 50 minutes in week 1 to 100 minutes in week 2. By the third week, they aimed to reach their walking goal of 30 minutes most days of the week, totaling at least 150 minutes of moderate PA per week. The control group was not provided with specific PA recommendations but wasn't restricted from engaging in PA during pregnancy. |
| Nobles 2015 | The overall goal of the exercise intervention was to encourage pregnant women to achieve the College’s guidelines for physical activity during pregnancy; that is, 30 minutes or more of moderate-intensity physical activity on most days of the week.8 Specific activities were self-selected and included dancing, walking, and yard work. Weekly goals were to increase time spent in moderate intensity physical activity by 10% to safely progress toward the overall activity goal. Participants were provided a digital pedometer as a motivational tool and an activity diary to encourage self-monitoring. |
| Okido 2015 | Detailed description of "FITT-VP" components and an in-depth explanation of the exercise prescription implementation process. Pelvic floor muscle training (PFMT) |
| Oostdam 2012 | Women in the intervention group received an exercise programmer on 2 days of the week during the remaining duration of their pregnancy. Each exercise session lasted for 60 minutes. The exercise sessions consisted of aerobic and strength exercises aimed to control blood glucose levels. The training intensity was carefully and individually controlled. All exercise sessions were completed under the guidance and supervision of a specifically trained physiotherapist. The exercise sessions were located at the Department of Physiotherapy in the participating hospitals. |
| Pelaez 2019 | In addition to standard care, women in the intervention group engaged in a structured, supervised exercise program lasting at least 24 weeks, from weeks 12 to 36 of gestation. The program comprised 70 to 78 group sessions with 8-12 women per session, held three times per week, each lasting 60 to 65 minutes. These sessions took place in a hospital with controlled environmental conditions (altitude: 600 m; temperature: 19-21°C; humidity: 50%-60%). The intervention program adhered to the guidelines of the American College of Obstetricians and Gynecologists. During each session, participants utilized a heart rate monitor to maintain exercise intensity within the range of 65%-70% of their age-predicted maximum heart rate. To account for variations in maternal heart rate responses, the Borg Rating of Perceived Exertion Scale was also employed, with a target score of 12-14 ("somewhat hard"). |
| Price 2012 | The intervention consisted of supervised aerobic training lasting 45-60 minutes, conducted four times a week at moderate intensity (Borg Scale rating of 12-14), in accordance with the ACOG exercise guidelines. The program included a variety of activities, such as step aerobics, group walking over hilly terrain, and circuit training. The circuit training involved alternating between 1-10 minutes of aerobic exercise on various equipment (treadmills, elliptical trainers, stationary bicycles) and an equal duration of weight training using machines, with a weight allowing for one set of 20 repetitions. Upper extremity exercises included overhead press, seated bench press, seated rowing, pectoral flexion, triceps extension, and bicep curls. Lower extremity exercises involved leg extension and hip adduction/abduction. Core muscle exercises included back extension and loaded torso rotation using weight machines, followed by crunches and prone and supine bridges on exercise balls. Each circuit session concluded with 5 minutes of stretching for the hamstrings, quadriceps, and calf muscles. To achieve four exercise sessions weekly, each participant also engaged in an individual brisk 30- to 60-minute walk once a week. |
| Renault 2014-PA | Detailed description of "FITT-VP" components and an in-depth explanation of the exercise prescription implementation process. |
| Ruiz 2013 | The intervention included light- to moderate-intensity aerobic and resistance exercises performed 3 days a week (50-55 minutes per session). |
| Seneviratne 2015 | The intervention group engaged in a structured home-based antenatal exercise program, focusing on moderate-intensity cycling using magnetic stationary bicycles from 20 to 35 weeks of gestation. Participants received a written program outlining the frequency and duration of weekly exercises. They were also equipped with heart rate monitors to be worn during cycling sessions, with target heart rates to maintain moderate exercise intensity (40–59% VO2 reserve). Each exercise session encompassed a 5-minute warm-up and cool-down period at low intensity. A total of 67 exercise sessions were prescribed, with frequency varying between three and five sessions per week and exercise duration ranging from 15 to 30 minutes per session based on the stage of pregnancy. An exercise physiologist visited each participant's home at the start of the intervention to provide assistance with exercise-related issues. Data on the number of sessions completed, exercise duration, and intensity were collected by downloading heart rate monitor data using POLAR PROTRAINER 5 software. The control group did not receive a prescribed exercise intervention or heart rate monitors. |
| Simmons 2015 | The effectiveness of the physical activity (PA) intervention was assessed using the Pregnancy Physical Activity Questionnaire, which included 32 activities encompassing household/caregiving, occupational, sports/exercise, and inactivity during the current trimester. Respondents could add activities not listed. In the DALI study, two questions related to cycling to work (4.0 metabolic equivalent of task or MET) and cycling for fun or exercise (8.0 MET) were added due to their frequent performance in some European countries. Participants selected the category that best represented the time spent in these activities, and their duration was multiplied by intensity, resulting in an average weekly energy expenditure (MET hours per week) for each activity. Open-ended activities were assigned MET values using a compendium of physical activities to categorize them by intensity (sedentary, light, moderate, vigorous). Self-reported moderate and vigorous activities were combined and presented as MET hours per week of moderate-to-vigorous physical activity (MVPA). |
| Simmons 2017 | The message delivery was based on principles of patient empowerment and cognitive behavioral techniques influenced by Motivational Interviewing. The intervention involved five face-to-face sessions lasting approximately 30-45 minutes each, along with up to four telephone calls or email contacts. Face-to-face sessions primarily took place in hospital or midwife practices. At least four coaching sessions were expected to occur before the second measurement session at 24-28 weeks, and the intervention was completed by 35 weeks of gestation. The effectiveness of the physical activity intervention was evaluated using the Pregnancy Physical Activity Questionnaire (PPAQ) at three time points. The PPAQ originally included 32 activities related to household/caregiving, occupational, sports/exercise, and inactivity during the current trimester. Open-ended questions allowed participants to add activities not listed. In the DALI study, two questions regarding cycling to work (4.0 MET) and cycling for fun or exercise (8.0 MET) were added due to their frequent occurrence in some European countries. Participants selected the category that best represented the time spent on these activities, and their duration was multiplied by intensity to calculate the average weekly energy expenditure (MET hours/week) for each activity. A compendium of physical activities was used to obtain MET values for open-ended reported activities, categorized by intensity (sedentary, light, moderate, vigorous). Nutrition was assessed using a customized short food frequency questionnaire covering key foods related to the intervention messages and calculated based on frequency and portion size. |
| Tomić V 2013 | The study group was engaged in regular aerobic exercise that consisted of a warm-up period (5 minutes), aerobic exercise (30 minutes), stretching (10 minutes), and the cooldown period (5 minutes). Exercise was performed 3 times per week during the whole pregnancy period. All sessions were supervised by an expert kinesiologist. The exercise program and intensity were in compliance with the guidelines set by the ACOG and the ACSM. |
| Uria-M 2022 | Women assigned to the intervention group received standard obstetric attention and a structured and supervised online moderate exercise intervention program three days a week (50–60 min/session) from the 8–10th week of pregnancy to the end of the third trimester, at the 38–39th week. Of the three sessions, two were carried out via Zoom 120 and the last session was carried out following YouTube videos, which had been previously 121 filmed by the physical activity professionals in our research group. The content of 122 the videos on YouTube were adapted to the weeks of pregnancy. |
| Ussher 2015 | There were 14 sessions of supervised exercise, twice a week for 6 weeks (one session with behavioral support for smoking cessation) and then weekly for 2 weeks. Following a familiarization session at the first visit, participants were advised to aim for 30 minutes of continuous treadmill walking during each session. Following guidelines,58 moderate-intensity exercise was prescribed according to age and current activity levels and was monitored using a polar heart-rate monitor. The intensity of exercise was also guided by a rating of perceived exertion59 (‘fairly light’ to ‘somewhat hard’) and by the ‘talk test’. |
| Wang 2016 | Stationary cycling program：Detailed description of "FITT-VP" components and an in-depth explanation of the exercise prescription implementation process. |
| Zhao 2022 | The experimental group received exercise intervention in a dedicated room for pregnant women, led by a team comprising the researcher, a nutritionist, and a sports medicine expert. Participants in the study group were required to complete exercise sessions at least three times a week for a minimum of 6 weeks, totaling at least 18 activities. To ensure moderate exercise intensity, heart rate was monitored using an exercise bracelet, and the Borg subjective physical sensation scale was used to assess the perception of effort. Exercise duration adhered to the American College of Obstetricians and Gynecologists (ACOG) guidelines for pregnancy, lasting 50-60 minutes. The intervention involved resistance exercises for upper and lower limb muscles, covering six body regions. Sports medicine experts provided guidance and instruction during the exercises, which concluded with a 5-minute stretching routine and relaxation. Patients commenced the exercise plan 1-2 hours after consuming staple food to prevent hypoglycemia, and any participants experiencing discomfort or adverse symptoms during the intervention were closely observed and promptly treated to ensure their safety. |
| Jin 2022 | Participants in the experimental group engaged in the Gymnastics for Pregnant Women program with face-to-face guidance. They completed this program 10 times per week from enrollment and were encouraged to continue throughout pregnancy while receiving standard perinatal care for gestational diabetes mellitus. The program, designed by clinical obstetricians and exercise specialists, consisted of 15-minute sessions with eight sections: Warm-up, Finger stretch, Arm stretch, fluttering of arms like butterflies, stretching like a bird in flight, swinging arms in bow step, Waving arms in the “Lucky Cat” style, and Cool down. It combined resistance and aerobic exercises, focusing on the upper and lower limbs, while incorporating some aerobic activity. Preliminary results from a 23-person experiment showed that the Gymnastics for Pregnant Women program led to lower postprandial blood glucose levels in the experimental group compared to the control group after 2 weeks of intervention, indicating its effectiveness. Over 90% of participants achieved the target heart rate, indicative of moderate-intensity physical activity, with no reported adverse events during the preliminary experiment. Participants recorded their blood glucose levels, heart rate during exercise, exercise duration and frequency, and reported results weekly through a WeChat group. Researchers offered recommendations and online guidance based on participants' information, including diet records, blood glucose measurements, and weight gain. |
| Gao 2019 | The detailed content of "FITT-VP" was not described, and the process of implementing the exercise prescription was introduced. |
| Kokic 2018 | Women in the Exercise Group (EG) initiated an individualized, structured exercise program twice a week in addition to their standard prenatal care, accompanied by a daily requirement of at least 30 minutes of brisk walking. This exercise regimen commenced upon GDM diagnosis and persisted throughout pregnancy. Attendance was meticulously recorded, and participants were tasked with maintaining a diary of their daily walks. The program duration was set at a minimum of 6 weeks, with an expected attendance rate of at least 70% of calculated exercise sessions until the 38th week of pregnancy. The Control Group (CG) received standard prenatal care for GDM without discouragement from engaging in self-directed exercise.  Each exercise session spanned 50–55 minutes and encompassed aerobic exercise (20 minutes), resistance exercises (20–25 minutes), pelvic floor and stretching exercises, concluding with a 10-minute relaxation period. The aerobic segment utilized a treadmill to achieve a heart rate within the aerobic zone (65–75% of maximum heart rate), targeting values of 13–14 on the Borg Rating of Perceived Exertion scale. Women could adjust treadmill parameters to meet intensity goals. Continuous monitoring of maternal heart rate was conducted using Mio Alpha. Resistance exercises covered major muscle groups with three sets of 10–15 repetitions each, following the Borg scale target values from the aerobic segment. Standardized resistance exercise protocols included activities for the trunk, upper and lower limbs, utilizing body weight, elastic bands, and handheld weights. Stretching, pelvic floor exercises, and a brief relaxation period concluded each session.  Participants in both groups adhered to medical nutrition therapy for GDM, with a daily intake of 1800 kcal, distributed across three main meals and three snacks, comprising 20% proteins (90 g), 30% fat (60 g), and 50% carbohydrates (225 g). |
| Halse 2015 | Supervised home-based exercise program. As detailed previously, mean compliance to the supervised cycling program was 96%, with a mean of 1±1 supervised sessions missed over the duration of the intervention for each woman. No adverse effects were reported in response to the exercise intervention. Participants cycled a mean of 10.0±1.3 km during the first week of the program (exercise duration, 25–30 min), progressing to 14.8±1.4 km during the last week of the program (40–45 min) (P G 0.001). Mean maternal HR during the conditioning phase of each session was 138±9 bpm (74%±4% of age-predicted HRmax) and mean RPE was 14 ± 1, indicating the exercise to be perceived as “somewhat hard”. These variables decreased during the 5-min recovery pedaling to a HR of 115±8 bpm and an RPE of 11±2 (fairly light), respectively. |
| Wu 2022 | A multifaceted quantitative exercise intervention model was developed to enhance compliance with exercise therapy among GDM patients. The model was created collaboratively by obstetricians, dietitians, diabetes nurses, psychological counselors, and exercise therapists with extensive clinical experience. It involved evaluating patients' body weight, exercise indications, job type, exercise habits, and hobbies, primarily advocating aerobic exercises combined with resistance exercises. Specific activity intensities were assigned using established systems, and patients were encouraged to engage in moderate-intensity exercises for at least 30 minutes per day and 150 minutes per week, with no more than three days between sessions. Implementation included structured group courses, audio-visual materials for practical guidance, individualized exercise recommendations, and regular follow-up through a WeChat communication platform. The goal was to provide comprehensive support and guidance for GDM patients in their exercise therapy journey. |
| Wein 1999 | Participants in the study completed a questionnaire detailing their diet and exercise history. The diet was assessed based on the fat, residue, and sugar content, with scores ranging from 1 to 3 (indicating poor to good), and a total score was computed. Exercise levels were scored on a scale of 0 to 7, ranging from totally sedentary (0) to athletic training (7). Follow-up testing and questionnaires were administered in the same manner during repeat glucose tolerance test visits. |
| Tandon 2022 | Stationary cycling program：Detailed description of "FITT-VP" components and an in-depth explanation of the exercise prescription implementation process. |
| Rather 2008 | Stationary cycling program：Detailed description of "FITT-VP" components and an in-depth explanation of the exercise prescription implementation process. |
| Cheung 2011 | This 12-month randomized controlled trial (RCT) involved measurements of weight, height, and a glucose tolerance test before randomizing subjects into Intervention or Control groups. Both groups received basic advice on dietary and physical activity (PA) guidelines. The Intervention group underwent patient-centered counseling and self-management education to modify PA behavior. The adoption phase (0–6 months) focused on cognitive determinants and goal-setting using pedometers. Scripted phone calls were made at 2, 6, and 10 weeks. The maintenance phase (6–12 months) emphasized self-directed strategies, with scripted calls at 26 and 34 weeks, and postcards reinforcing behavior change were mailed. The Intervention aimed for 30 minutes of moderate-intensity activity on 5 days or 10,000 steps/day on 5 days/week. Evaluation pedometers were locked to prevent monitoring and adjustment of activity. |

**NOTE:** “FITT-VP” stands for the components of a comprehensive exercise prescription framework: **Frequency (F):** How often the exercise is performed. **Intensity (I):** The level of difficulty or effort of the exercise. **Time (T):** The duration or time spent on each exercise session. **Type (T):** The specific kind or mode of exercise. **Volume (V):** The total amount of work or exercise performed. **Progression (P):** How the exercise program advances over time to maintain or enhance its effectiveness. This framework is commonly used in exercise prescription to ensure that physical activity is tailored to an individual's needs and abilities.

**Table 2** **The inclusion study summarized the implementation strategies for managing GDM at different stages of exercise intervention.**

| GDM Management Stage | Intervention Time | Exercise Prescription Basis | Face-to-Face Supervision | Intervention Location |
| --- | --- | --- | --- | --- |
| GDM Prevention (27 Rcts) | Early pregnancy to Mid-pregnancy | ACOG、previous study | Yes | Health Care Center、Hospitals、Home |
| GDM Treatment (6 Rcts) | Mid-pregnancy to late pregnancy | ACOG、qualified fitness specialist | Yes | Health Care Center、Hospitals、Home |
| GDM Prognosis (4 Rcts) | After childbirth for several years | NA | No | Home |
