# Supplementary material for: Comprehensive management of gestational diabetes mellitus: practical efficacy of exercise therapy and sustained intervention strategies
Source: Front Endocrinol (Lausanne). 2024 Oct 3;15:1347754. doi: 10.3389/fendo.2024.1347754 (PMC11484007; doi:10.3389/fendo.2024.1347754)
Supplement: ADDITIONAL FILE 1 — Search Strategy. [file DataSheet1.zip › Additional file 1.DOCX]

**Additional file 1: Search strategy**

**As an example, using the following four databases:**

**Box 1. PubMed Search Strategy**

((Diabetes, Gestational OR Diabetes, Pregnancy-Induced OR Diabetes, Pregnancy Induced OR Pregnancy-Induced Diabetes OR Gestational Diabetes OR Diabetes Mellitus, Gestational OR Gestational Diabetes Mellitus OR Diabetes, Pregnancy-Induced OR Gestational Diabetes OR Pregnancy-Induced Diabetes OR Gestational Diabetes Mellitus OR Diabetes, Pregnancy Induced OR Diabetes Mellitus, Gestational) AND (Exercise OR Exercises OR Physical Activity OR Activities, Physical OR Activity, Physical OR Physical Activities OR Exercise, Physical OR Exercises, Physical OR Physical Exercise OR Physical Exercises OR Acute Exercise OR Acute Exercises OR Exercise, Acute OR Exercises, Acute OR Exercise, Isometric OR Exercises, Isometric OR Isometric Exercises OR Isometric Exercise OR Exercise, Aerobic OR Aerobic Exercise OR Aerobic Exercises OR Exercises, Aerobic OR Exercise Training OR Exercise Trainings OR Training, Exercise OR Trainings, Exercise)) AND (Effectiveness OR program evaluation OR randomized controlled trial OR RCT OR controlled trial OR trial)

**Box 2. Cochrane Library Search Strategy**

Date Run: 21/11/2023 17:52:39

Comment:

ID Search Hits

#1 Diabetes, Gestational OR Diabetes, Pregnancy-Induced OR Diabetes, Pregnancy Induced OR Pregnancy-Induced Diabetes OR Gestational Diabetes OR Diabetes Mellitus, Gestational OR Gestational Diabetes Mellitus OR Diabetes, Pregnancy-Induced OR Gestational Diabetes OR Pregnancy-Induced Diabetes OR Gestational Diabetes Mellitus OR Diabetes, Pregnancy Induced OR Diabetes Mellitus, Gestational 5782

#2 Exercise OR Exercises OR Physical Activity OR Activities, Physical OR Activity, Physical OR Physical Activities OR Exercise, Physical OR Exercises, Physical OR Physical Exercise OR Physical Exercises OR Acute Exercise OR Acute Exercises OR Exercise, Acute OR Exercises, Acute OR Exercise, Isometric OR Exercises, Isometric OR Isometric Exercises OR Isometric Exercise OR Exercise, Aerobic OR Aerobic Exercise OR Aerobic Exercises OR Exercises, Aerobic OR Exercise Training OR Exercise Trainings OR Training, Exercise OR Trainings, Exercise 177312

#3 Effectiveness OR program evaluation OR randomized controlled trial OR RCT OR controlled trial OR trial 1422420

#4 #1 AND #2 AND #3 1177

**Box 3. Web of Science Search Strategy**

(((TI=(Diabetes, Gestational)) OR TI=(Diabetes, Pregnancy-Induced OR Diabetes, Pregnancy Induced OR Pregnancy-Induced Diabetes OR Gestational Diabetes OR Diabetes Mellitus, Gestational OR Gestational Diabetes Mellitus)) AND ALL=(Exercises OR Physical Activity OR Activities, Physical OR Activity, Physical OR Physical Activities OR Exercise, Physical OR Exercises, Physical OR Physical Exercise OR Physical Exercises OR Acute Exercise OR Acute Exercises OR Exercise, Acute OR Exercises, Acute OR Exercise, Isometric OR Exercises, Isometric OR Isometric Exercises OR Isometric Exercise OR Exercise, Aerobic OR Aerobic Exercise OR Aerobic Exercises OR Exercises, Aerobic OR Exercise Training OR Exercise Trainings OR Training, Exercise OR Trainings, Exercise ))

**Box 4. Search strategy MEDLINE.**

| **Number** | **Search terms** |
| --- | --- |
| 1 | aerobic |
| 2 | sport |
| 3 | exercise |
| 4 | training |
| 5 | physical exercise |
| 6 | physical activity |
| 7 | lifestyle |
| 8 | #1 OR #2 OR #3 OR #4 OR #5 OR #6 OR #7 |
| 9 | gestational diabetes mellitus |
| 10 | diabetes |
| 11 | pregnancy induced |
| 12 | pregnancy-induced diabetes |
| 13 | pregnancy-induced |
| 14 | gestational diabetes mellitus |
| 15 | diabetes mellitus, gestational |
| 16 | diabetes mellitus |
| 17 | #9 OR #10 OR #11 OR #12 OR #13OR #14 OR #15 OR #16 |
| 18 | Type 2 |
| 19 | slow-onset diabetes mellitus |
| 20 | type 2 diabetes mellitus |
| 21 | type 2 diabetes |
| 22 | stable diabetes mellitus |
| 23 | maturity onset diabetes mellitus |
| 24 | adult-onset diabetes mellitus |
| 25 | #18 OR #19 OR #20 OR #21 OR #22OR #23 OR #24 |
| 26 | effectiveness |
| 27 | program evaluation |
| 28 | randomized controlled trial |
| 29 | RCT |
| 30 | #26 OR #27 OR #28 OR #29 |
| 31 | #8AND #17 OR #25 AND #30 |
