# Supplementary material for: Comprehensive management of gestational diabetes mellitus: practical efficacy of exercise therapy and sustained intervention strategies
Source: Front Endocrinol (Lausanne). 2024 Oct 3;15:1347754. doi: 10.3389/fendo.2024.1347754 (PMC11484007; doi:10.3389/fendo.2024.1347754)
Supplement: ADDITIONAL FILE 1 — Search Strategy. [file DataSheet1.zip › Additional file 6 (Review protocol).PDF]

Systematic review

A list of fields that can be edited in an update can be found here

1. * Review title.

Give the title of the review in English

Exercise Therapy for GDM and Post-GDM Type 2 Diabetes: A Systematic Review and Meta-analysis

2. Original language title.

For reviews in languages other than English, give the title in the original language. This will be displayed with the English language title.

Exercise Therapy for GDM and Post-GDM Type 2 Diabetes: A Systematic Review and Meta-analysis

3. * Anticipated or actual start date.

Give the date the systematic review started or is expected to start.

27/09/2023

4. * Anticipated completion date.

Give the date by which the review is expected to be completed.

24/12/2023

5. * Stage of review at time of this submission.

This field uses answers to initial screening questions. It cannot be edited until after registration.

Tick the boxes to show which review tasks have been started and which have been completed.

Update this field each time any amendments are made to a published record.

The review has not yet started: Yes

Review stage	Started	Completed
Preliminary searches	No	No
Piloting of the study selection process	No	No
Formal screening of search results against eligibility criteria	No	No
Data extraction	No	No
Risk of bias (quality) assessment	No	No
Data analysis	No	No

Provide any other relevant information about the stage of the review here.

6. * Named contact.

The named contact is the guarantor for the accuracy of the information in the register record. This may be any member of the review team.

Hao Xu

Email salutation (e.g. "Dr Smith" or "Joanne") for correspondence:
Ms Xu

7. * Named contact email.

Give the electronic email address of the named contact.

megan.xu@foxmail.com

8. Named contact address

PLEASE NOTE this information will be published in the PROSPERO record so please do not enter private information, i.e. personal home address

Give the full institutional/organisational postal address for the named contact.

School of Physical Education, China University of Geosciences, Wuhan, 430074, China

9. Named contact phone number.

Give the telephone number for the named contact, including international dialling code.

13409699375

10. * Organisational affiliation of the review.

Full title of the organisational affiliations for this review and website address if available. This field may be completed as 'None' if the review is not affiliated to any organisation.

China University of Geosciences

Organisation web address:

11. * Review team members and their organisational affiliations.

Give the personal details and the organisational affiliations of each member of the review team. Affiliation refers to groups or organisations to which review team members belong.
NOTE: email and country now MUST be entered for each person, unless you are amending a published record.

Miss Hao Xu. China University of Geosciences (Wuhan)
Mr Renyi Liu. China University of Geosciences (Wuhan)

12. * Funding sources/sponsors.

Details of the individuals, organizations, groups, companies or other legal entities who have funded or sponsored the review.

None

Grant number(s)
State the funder, grant or award number and the date of award
None

13. * Conflicts of interest.

List actual or perceived conflicts of interest (financial or academic).

None

14. Collaborators.

Give the name and affiliation of any individuals or organisations who are working on the review but who are not listed as review team members. **NOTE: email and country must be completed for each person, unless you are amending a published record.**

15. * Review question.

State the review question(s) clearly and precisely. It may be appropriate to break very broad questions down into a series of related more specific questions. Questions may be framed or refined using PI(E)COS or similar where relevant.

The participants are normal pregnant women or GDM patients or women with a history of GDM. (P: participants);(2)The intervention type in the experimental group was exercise (I: interventions);(3)Perinatal women who received usual care or other therapies that do not involve physical activity intervention were as the control group (C: comparisons);(4)We have selected disease incidence rates and the results of the 75-gram Oral Glucose Tolerance Test (75g-OGTT) as the outcome data for this study. (O: outcomes);(5)The analysis type in the literature is a randomized controlled trial (RCT) (S: study design).

16. * Searches.

State the sources that will be searched (e.g. Medline). Give the search dates, and any restrictions (e.g. language or publication date). Do NOT enter the full search strategy (it may be provided as a link or attachment below.)

Search of the PubMed, Embase, Web of Science, Cochrane Library, MEDLINE, ScienceDirect (Elsevier) from inception to August 2023 for randomized controlled trials (RCTs) aimed at assessing the effect of exercise interventions on GDM and Type 2 Diabetes After GDM.

17. URL to search strategy.

Upload a file with your search strategy, or an example of a search strategy for a specific database, (including the keywords) in pdf or word format. In doing so you are consenting to the file being made publicly accessible.

Or provide a URL or link to the strategy. Do NOT provide links to your search **results**.

https://www.webofscience.com/wos/woscc/summary/0326c8e1-7343-4738-939b-1d71c8af2fac-a1cf2eeb/relevance/1

Yes I give permission for this file to be made publicly available

18. * Condition or domain being studied.

Give a short description of the disease, condition or healthcare domain being studied in your systematic review.

Ptimizing the Impact of Exercise Interventions in Gestational Diabetes: A Comprehensive Comparative Analysis and Identifying Enhancing Factors

19. * Participants/population.

Specify the participants or populations being studied in the review. The preferred format includes details of both inclusion and exclusion criteria.

The participants are women

20. * Intervention(s), exposure(s).

Give full and clear descriptions or definitions of the interventions or the exposures to be reviewed. The preferred format includes details of both inclusion and exclusion criteria.

Exercise

21. * Comparator(s)/control.

Where relevant, give details of the alternatives against which the intervention/exposure will be compared (e.g. another intervention or a non-exposed control group). The preferred format includes details of both inclusion and exclusion

criteria.

Usual care or other therapies that do not involve physical activity intervention were as the control group

22. * Types of study to be included.

Give details of the study designs (e.g. RCT) that are eligible for inclusion in the review. The preferred format includes both inclusion and exclusion criteria. If there are no restrictions on the types of study, this should be stated.

RCT

23. Context.

Give summary details of the setting or other relevant characteristics, which help define the inclusion or exclusion criteria.

Gestational diabetes mellitus affects millions of pregnant women. Lifestyle intervention is recommended as the first-line treatment, in which exercise plays an important role. Effective and safe exercise is required to facilitate glycaemic control and improve the sequelae resulting from abnormal glucose metabolism due to GDM.

24. * Main outcome(s).

Give the pre-specified main (most important) outcomes of the review, including details of how the outcome is defined and measured and when these measurement are made, if these are part of the review inclusion criteria.

The mean and standard deviation of the 75g-OGTT in the experimental and control groups following exercise intervention, as well as the incidence data of GDM and type 2 diabetes after GDM.

Measures of effect
The category of Continuous data, we calculated the effect sizes (MD) and their 95% confidence intervals for the experimental and control groups in each study. The outcome regarding the incidence rates of GDM and type 2 diabetes after GDM was Dichotomous (Binary) data. We used Relative Risk (RR) to compare the differences in the incidence rates of gestational diabetes and type 2 diabetes after GDM between the exercise group and the control group.

25. * Additional outcome(s).

List the pre-specified additional outcomes of the review, with a similar level of detail to that required for main outcomes. Where there are no additional outcomes please state ‘None’ or ‘Not applicable’ as appropriate to the review

None

Measures of effect
None

26. * Data extraction (selection and coding).

Describe how studies will be selected for inclusion. State what data will be extracted or obtained. State how this will be done and recorded.

Inclusion criteria: (based on the PICOS (1) The participants are women. (P: participants); (2) The intervention type in the experimental group was exercise (I: interventions); (3) Perinatal women who received usual care or other therapies that do not involve physical activity intervention were as the control group (C: comparisons); (4) We have selected disease incidence rates and the results of the 75-gram Oral Glucose Tolerance Test (75g-OGTT) as the outcome data for this study. (O: outcomes); (5) The analysis type in the literature is a randomized controlled trial (RCT) (S: study design).

27. * Risk of bias (quality) assessment.

State which characteristics of the studies will be assessed and/or any formal risk of bias/quality assessment tools that will be used.

According to the preliminary risk assessment for publication bias as recommended by the Cochrane Collaboration.

28. * Strategy for data synthesis.

Describe the methods you plan to use to synthesise data. This **must not be generic text** but should be **specific to your review** and describe how the proposed approach will be applied to your data.
If meta-analysis is planned, describe the models to be used, methods to explore statistical heterogeneity, and software package to be used.

The mean and standard deviation of the 75g-OGTT in the experimental and control groups following exercise intervention, as well as the incidence data of GDM and type 2 diabetes after GDM, were analyzed using RevMan 5.4 software. The 75g-OGTT is a clinical laboratory examination employed to assess an individual’s blood sugar metabolism. This test entails the oral consumption of a 75-gram glucose solution while the individual is in a fasting state. Subsequently, blood glucose levels are measured at various time intervals, typically including 0 hours (fasting), 1 hour, and 2 hours after glucose ingestion. The above fell into the category of Continuous data, we calculated the effect sizes (MD) and their 95% confidence intervals for the experimental and control groups in each study. By aggregating all results from RCTs included in the meta-analysis, we could determine whether there was an improvement in fasting blood glucose and 2 hours after glucose ingestion. This determination depended on whether there was a significant difference in the outcomes between the experimental and control groups. If MD was positive, it indicated that the mean of the experimental group was greater than that of the control group, and if MD was negative, it indicated that the control group’s mean was greater than that of the experimental group. The outcome regarding the incidence rates of GDM and type 2 diabetes after GDM was Dichotomous (Binary) data. We used Relative Risk (RR) to compare the differences in the incidence rates of gestational diabetes and type 2 diabetes after GDM between the exercise group and the control group. If RR equaled 1, then the incidence rates in both groups were equal; if RR was greater than 1, it indicated a higher incidence rate in the exercise group, and if RR was less than 1, it indicated a lower incidence rate in the exercise group. Whether it was Continuous outcomes or Dichotomous outcomes, if I^2 was less than or equal to 50% or the p-value was greater than 0.05, indicating low heterogeneity, a fixed-effects model was applied. Conversely, if I^2 was greater than 50% or the p-value was less than or equal to 0.05, indicating high heterogeneity.

29. * Analysis of subgroups or subsets.

State any planned investigation of ‘subgroups’. Be clear and specific about which type of study or participant will be included in each group or covariate investigated. State the planned analytic approach.

I do not have plans for subgroup analysis at this time

30. * Type and method of review.

Select the type of review, review method and health area from the lists below.

Type of review

Cost effectiveness	No
Diagnostic	No
Epidemiologic	No
Individual patient data (IPD) meta-analysis	No
Intervention	No
Living systematic review	No
Meta-analysis	Yes
Methodology	No
Narrative synthesis	No
Network meta-analysis	No
Pre-clinical	No
Prevention	No
Prognostic	No
Prospective meta-analysis (PMA)	No
Review of reviews	No

Service delivery	No
Synthesis of qualitative studies	No
Systematic review	Yes
Other	No

Health area of the review

Alcohol/substance misuse/abuse	No
Blood and immune system	No
Cancer	No
Cardiovascular	No
Care of the elderly	No
Child health	No
Complementary therapies	No
COVID-19	No
Crime and justice	No
Dental	No
Digestive system	No
Ear, nose and throat	No
Education	No
Endocrine and metabolic disorders	No
Eye disorders	No
General interest	No
Genetics	No
Health inequalities/health equity	No
Infections and infestations	No
International development	No
Mental health and behavioural conditions	No
Musculoskeletal	No
Neurological	No
Nursing	No
Obstetrics and gynaecology	Yes

Oral health	No
Palliative care	No
Perioperative care	No
Physiotherapy	No
Pregnancy and childbirth	Yes
Public health (including social determinants of health)	Yes
Rehabilitation	No
Respiratory disorders	No
Service delivery	No
Skin disorders	No
Social care	No
Surgery	No
Tropical Medicine	No
Urological	No
Wounds, injuries and accidents	No
Violence and abuse	No

31. Language.

Select each language individually to add it to the list below, use the bin icon to remove any added in error.

English

There is an English language summary.

32. * Country.

Select the country in which the review is being carried out. For multi-national collaborations select all the countries involved.

China

33. Other registration details.

Name any other organisation where the systematic review title or protocol is registered (e.g. Campbell, or The Joanna Briggs Institute) together with any unique identification number assigned by them.
If extracted data will be stored and made available through a repository such as the Systematic Review Data Repository (SRDR), details and a link should be included here. If none, leave blank.

34. Reference and/or URL for published protocol.

If the protocol for this review is published provide details (authors, title and journal details, preferably in Vancouver format)

No I do not make this file publicly available until the review is complete

35. Dissemination plans.

Do you intend to publish the review on completion?

No

36. Keywords.

Give words or phrases that best describe the review. Separate keywords with a semicolon or new line. Keywords help PROSPERO users find your review (keywords do not appear in the public record but are included in searches). Be as specific and precise as possible. Avoid acronyms and abbreviations unless these are in wide use.

Gestational Diabetes Mellitus、Type 2 Diabetes、 Exercise Prescription、Exercise Adherence、 Incidence Rate、Blood Glucose

37. Details of any existing review of the same topic by the same authors.

If you are registering an update of an existing review give details of the earlier versions and include a full bibliographic reference, if available.

38. * Current review status.

Update review status when the review is completed and when it is published.
New registrations must be ongoing so this field is not editable for initial submission.

Review_Ongoing

39. Any additional information.

Provide any other information relevant to the registration of this review.

40. Details of final report/publication(s) or preprints if available.

Leave empty until publication details are available OR you have a link to a preprint (NOTE: this field is not editable for initial submission).
List authors, title and journal details preferably in Vancouver format.
