# Supplementary figures and images for: Comprehensive management of gestational diabetes mellitus: practical efficacy of exercise therapy and sustained intervention strategies
Source: Front Endocrinol (Lausanne). 2024 Oct 3;15:1347754. doi: 10.3389/fendo.2024.1347754 (PMC11484007; doi:10.3389/fendo.2024.1347754)

**Additional file 4:** **Risk of bias.**

**
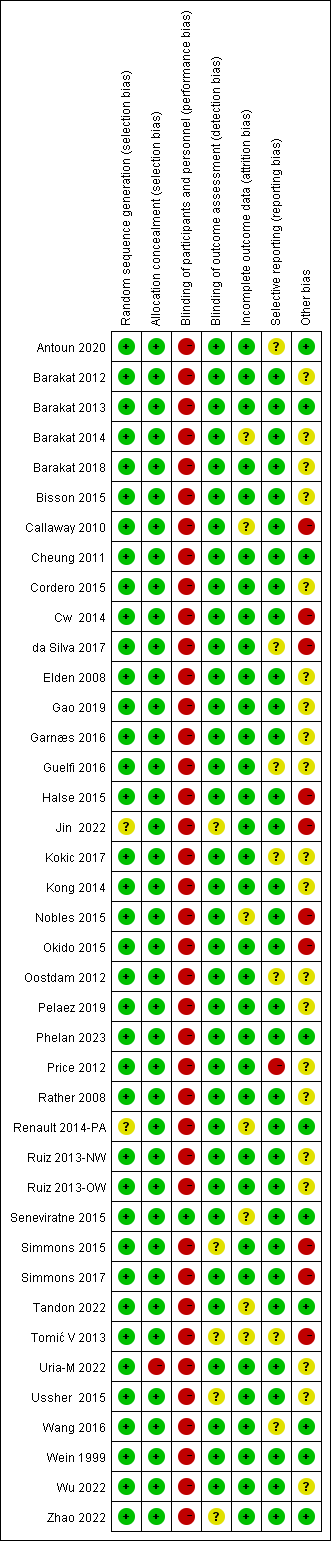
**

Supplement: ADDITIONAL FILE 1 — Search Strategy. [file DataSheet1.zip › Additional file 4.DOCX]
